# Theta-gamma coupling emerges from spatially heterogeneous cholinergic neuromodulation

**DOI:** 10.1371/journal.pcbi.1009235

**Published:** 2021-07-30

**Authors:** Yihao Yang, Howard Gritton, Martin Sarter, Sara J. Aton, Victoria Booth, Michal Zochowski

**Affiliations:** 1 Department of Physics, University of Michigan, Ann Arbor, Michigan, United States of America; 2 Department of Comparative Biosciences and Bioengineering, University of Illinois at Urbana-Champaign, Urbana, Illinois, United States of America; 3 Department of Psychology and Neuroscience Program, University of Michigan, Ann Arbor, Michigan, United States of America; 4 Department of Molecular, Cellular, and Developmental Biology, University of Michigan, Ann Arbor, Michigan, United States of America; 5 Departments of Mathematics and Anesthesiology, University of Michigan, Ann Arbor, Michigan, United States of America; 6 Department of Physics and Biophysics Program, University of Michigan, Ann Arbor, Michigan, United States of America; University of Pittsburgh, UNITED STATES

## Abstract

Theta and gamma rhythms and their cross-frequency coupling play critical roles in perception, attention, learning, and memory. Available data suggest that forebrain acetylcholine (ACh) signaling promotes theta-gamma coupling, although the mechanism has not been identified. Recent evidence suggests that cholinergic signaling is both temporally and spatially constrained, in contrast to the traditional notion of slow, spatially homogeneous, and diffuse neuromodulation. Here, we find that spatially constrained cholinergic stimulation can generate theta-modulated gamma rhythms. Using biophysically-based excitatory-inhibitory (E-I) neural network models, we simulate the effects of ACh on neural excitability by varying the conductance of a muscarinic receptor-regulated K^+^ current. In E-I networks with local excitatory connectivity and global inhibitory connectivity, we demonstrate that theta-gamma-coupled firing patterns emerge in ACh modulated network regions. Stable gamma-modulated firing arises within regions with high ACh signaling, while theta or mixed theta-gamma activity occurs at the peripheries of these regions. High gamma activity also alternates between different high-ACh regions, at theta frequency. Our results are the first to indicate a causal role for spatially heterogenous ACh signaling in the emergence of localized theta-gamma rhythmicity. Our findings also provide novel insights into mechanisms by which ACh signaling supports the brain region-specific attentional processing of sensory information.

## Author summary

Recent evidence indicating that ACh signaling is both transient and spatially circumscribed raises the question of how this feature impacts information processing in cortical networks. Here we demonstrate that spatially segregated ACh modulation of excitatory-inhibitory neural networks generates theta-modulated gamma rhythms, a hallmark of attention and information processing. Theta-gamma coupling arises naturally as neuronal activity traverses high-ACh regions of the network, and gamma activity alternates between distinct high-ACh sites at theta frequency. These findings provide novel insights into neurophysiological mechanisms for ACh-regulated theta-gamma coupling and uncoupling.

## Introduction

**Fig 1:**
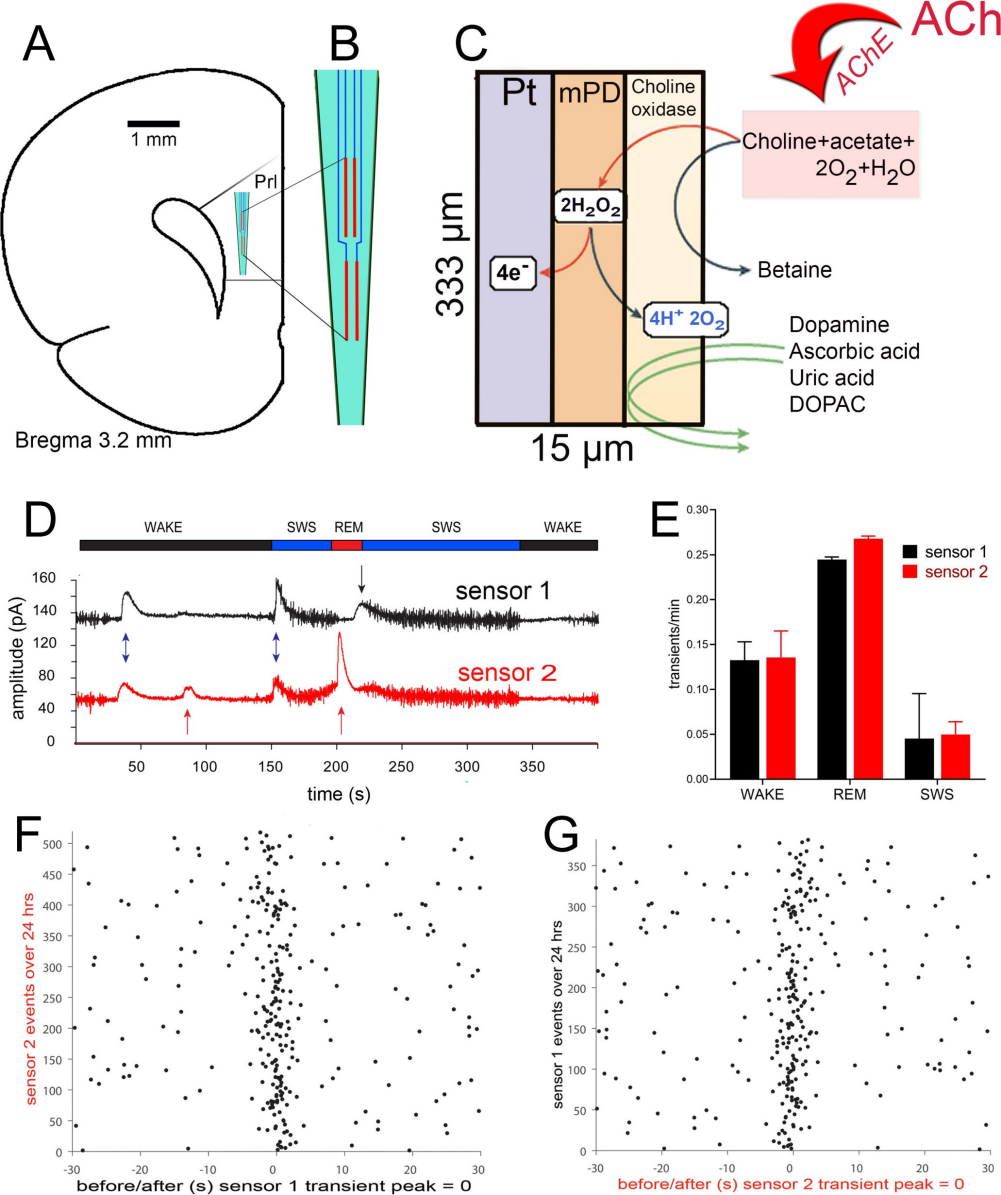
Spatial localization and asynchronicity of cholinergic signals. Cholinergic signaling is largely asynchronous and can influence target circuitry in a temporally and spatially highly heterogenous manner. Data recorded in prelimbic cortex (**A**) are shown here. The four platinum (Pt) recording sites fabricated onto a ceramic backbone electrode are illustrated in **B** and the placement of these recording sites in prelimbic cortex are shown in **A**. **C** depicts the dimensions of an individual recording site. The upper and lower pairs of recording sites were separated by 100 μm. The data shown in **D-G** were recorded via an upper sensor (“sensor 1) and a lower sensor (“sensor 2”). The neurochemical recording scheme, shown in C, was previously described in detail. Amperometric measures were validated in terms of reflecting newly released acetylcholine (ACh) release [10,52–54]. **D**. Fixed-potential amperometry signals from a representative animal during a 23.5-hour recording. Scored sleep states are identified above the raw amperometry signals shown below and include rapid eye movement (REM), Slow-wave sleep (SWS) and waking (WAKE) periods. Transients are denoted by arrows with unique transients shown in the same color as the corresponding trace and common transients shown as double blue arrows. **E**. Cholinergic transient event rates as a function of sleep state. Absolute event rates across the two sites show a high degree of similarity between one another. REM sleep states show the highest event rates/minute while SWS states show the fewest. **F, G.** Event rasters contrasting the timing of transient events for the two sites shown in d and e. Data from the opposite channel is shown relative to the onset of transient events for sensor 2 (**F**) or sensor 1 (**G**). A total of 524 events were detected from sensor 2 and 382 events were detected from sensor 1 in the 23.5-hour recording. Note that while the highest concentration of activity is coincident across the two sensors, only a fraction of each channels events occurred in close proximity (±2.5 s) to one another (sensor 1: 40.3%; sensor 2: 29.9%).

Acetylcholine (ACh) signaling in neocortex emanates from the basal forebrain (BF). Recent anatomical studies indicate that in contrast to more traditional views of the BF projection system as “diffusely” organized, afferent and efferent projections of the BF ACh system are highly topographically organized [1–4]. Prior notions of BF ACh activity as having relatively low temporal resolution and spatial selectivity, and characterized by predominantly extra-synaptic actions (i.e., “volume” transmission) [5–8], have also been refuted by more recent evidence indicating fast and spatially discrete ACh spread [8,9]. As a functional corollary of these developments, the prior conceptualization of ACh as acting diffusely and globally has been challenged by studies indicating event- or task trial-specific ACh signaling in specific neocortical regions [8,10]. Fig 1 shows data from multiple electrochemical recording sites in proximity, demonstrating asynchronous, spatially segregated neocortical ACh signaling.

Here, we investigate in silico the effects of temporally static but spatially circumscribed, heterogeneous activation of muscarinic ACh receptors on the activity patterns of excitatory-inhibitory (E-I) neural networks. Our simulation results indicate that localized theta (∼ 5 − 10Hz) and gamma (∼ 30 − 100Hz) band activity rhythms emerge in response to spatially segregated ACh modulation of neural excitability. Here, the modeled spatial cholinergic distributions are meant to represent a short snapshot from the evidence of spatially circumscribed ACh signaling in recording studies in rodents (Fig 1D), where discrete locations of high levels of cholinergic signaling were observed adjacent to locations with low levels of cholinergic activity. We analyzed the emerging neuronal activity patterns in the presence of stationary high levels of cholinergic signaling in a single versus in multiple locations of the network. Localized, gamma band activity rhythms emerged in cells undergoing high levels of cholinergic stimulation. Moreover, for multiple high-ACh ‘hotspots’, these gamma oscillations appeared only within the currently active network regions, resulting in their modulation at theta frequency.

Our results postulate that theta-gamma coupling is an emergent property of spatially segregated ACh modulation of neural response properties. We further identified mechanisms underlying the dependence of theta-gamma coupled activity on the spatial distribution of simulated ACh neuromodulation. In particular, gamma-band activity was supported in high-ACh regions via the pyramidal-interneuron gamma (PING) mechanism [11], where inhibitory interneurons strongly modulate and synchronize activity of pyramidal cells [11,12]. Theta band modulation of gamma activity within or between high-ACh regions was associated with spike frequency adaptation, linked to effects of muscarinic receptor activation on M-type K^+^ currents [13]. These mechanisms led to intrinsically tight coupling between gamma and theta band activity where the degree of theta-gamma coupling correlated with proximity to high-ACh regions. Additionally, we investigated the consequences of spatially heterogeneous ACh modulation on the attentional processing of external (sensory) stimuli.

Theta-gamma coupled activity in cortical and hippocampal areas is thought to be a hallmark of attentive cognitive processing [14] and multiple experimental studies have shown that ACh signaling promotes theta-gamma coupling in these circuits [15,16] (see Discussion). Our modeling results propose that this cognitively significant firing pattern is directly caused by spatially heterogeneous modulation of neural properties due to spatially circumscribed release of ACh.

## Results

New experimental data indicates that cholinergic signaling is largely asynchronous and can influence target circuitry in a temporally and spatially highly heterogenous manner. An example of such evidence is presented on Fig 1. In this experiment four platinum recording sites were fabricated onto a ceramic backbone electrode where the upper and lower pairs of recording sites were separated by 100 μm (Fig 1A). Fig 1D depicts sample measurements (in terms of currents) depicting localized temporal changes of ACh concentrations. Further analysis of these transients indicates that, while their overall number follows standard notion that the highest ACh release is during REM and the lowest happens during the SWS (Fig 1E), this release is localized and highly asynchronous (Fig 1F and 1G).

Here we elucidated, using in silico modeling, the dynamical changes in neuronal activity patterns stemming from such a localized and asynchronous mode of ACh signaling. Namely, we investigated how spatially localized regions of ACh neuromodulation generated network-wide oscillatory activity in the gamma and theta frequency bands, in two-dimensional E-I networks. Using Hodgkin-Huxley type model neurons, ACh effects on the slow, adapting M-type K^+^ current were simulated by varying its maximal conductance, $g_{K_{s}}$, in individual cells across the network. Through the action of muscarinic receptors, ACh blocks the M-type K^+^ current, thus low values of $g_{K_{s}}$ corresponded to high ACh modulation. Spatially heterogeneous ACh modulation was achieved by constructing spatial mappings of $g_{K_{s}}$ values for individual cells in the network. The $g_{K_{s}}$ map mimicked the post-synaptic effects of spatially localized, asynchronous transients of ACh release in a short time window (as suggested by results shown in Fig 1). To this end, we postulate that relevant network dynamics occur on two separate temporal scales: 1) fast dynamics on a timescale of milliseconds associated with neuronal firing, and 2) slow timescales on the order of 5–10 s associated with localized ACh release and subsequent degradation or uptake. Here, for simplicity, we investigated fast scale neuronal dynamics in the presence of fixed spatial distributions of ACh. In the $g_{K_{s}}$ maps, each unit square corresponded to a single cell in the model network (i.e. the unit length, being the side of the square, corresponded to the minimal distance between modeled cells), and the dimensions of $g_{K_{s}}$ modulated regions we consider encompass, on average, tens of neurons.

With network connectivity fixed in a local excitation/global inhibition topology, we show that different $g_{K_{s}}$ spatial mappings, which affect the excitability of both E and I cells, result in gamma and theta band rhythmic activity. While there is evidence that the M-current is present not only in excitatory pyramidal cells, but also in various classes of inhibitory interneurons, namely somatostatin positive (SST+) neurons [17], we obtained qualitatively similar results when neuromodulatory effects of the M-current were included only in excitatory neurons (S1 Fig). Consistent with previous work [11,12], continuously active E and I cells in regions of low $g_{K_{s}}$ values, corresponding to high ACh modulation, show gamma band oscillatory activity. Here, theta band modulation of gamma activity is an emergent property of the network, generated as firing activity traverses within or between spatially segregated low $g_{K_{s}}$ regions. Below, we first illustrate this novel mechanism for theta-gamma coupling with a randomly generated, spatial $g_{K_{s}}$ mapping and then analyze the mechanism in more detail for simple spatial $g_{K_{s}}$ mappings.

### Coexistence of theta/gamma rhythms is caused by spatially segregated $g_{K_{s}}$ distributions

**Fig 2:**
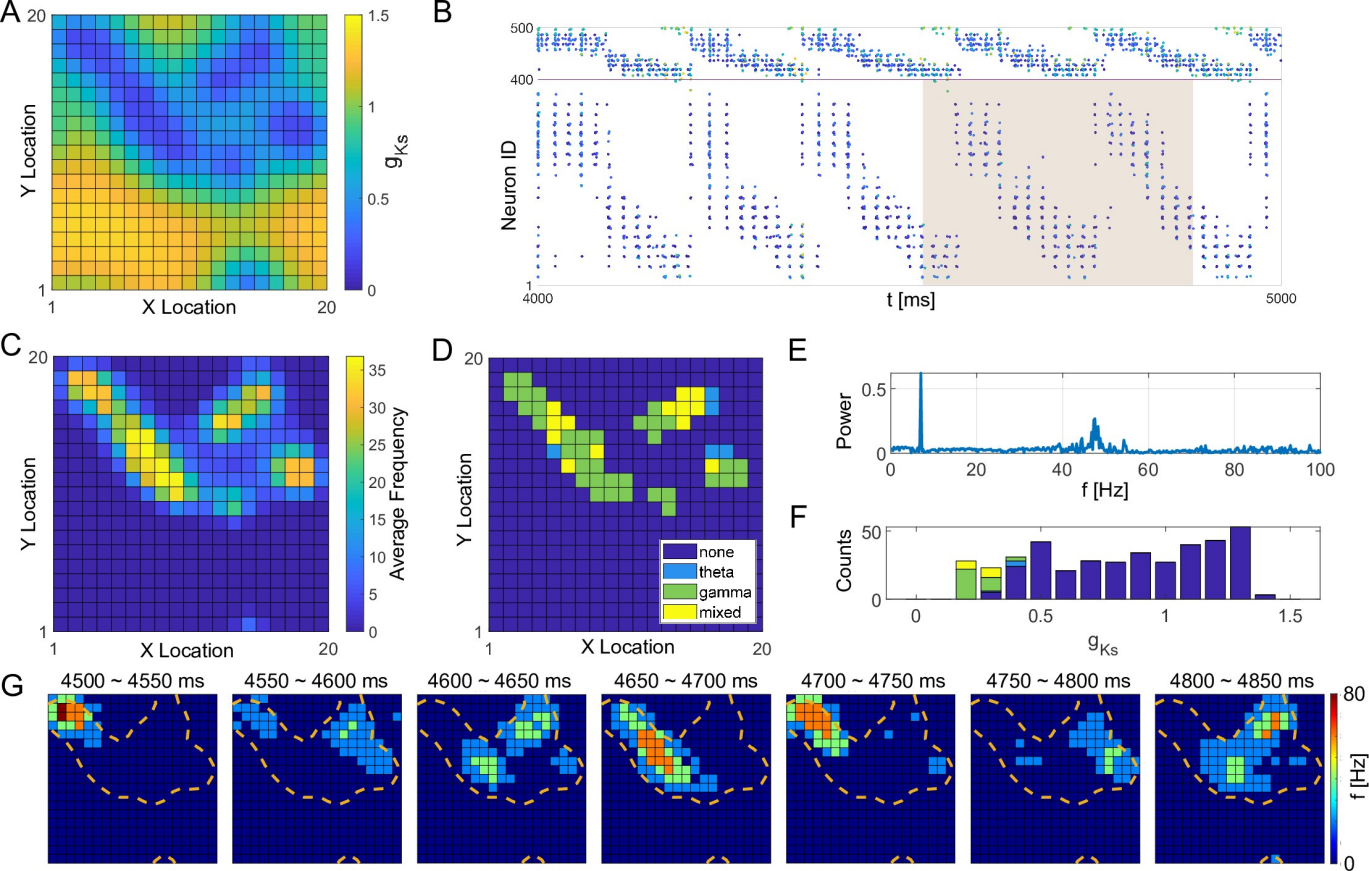
Theta and gamma band activity generated by a spatially heterogeneous $g_{K_{s}}$ distribution. **A,** Randomly generated spatial map of $g_{K_{s}}$ values on the 20×20 E cell lattice. **B,** Spike raster plot illustrating portion of E cell (cells 1−400) and I cell (cells 401−500) firing patterns. The pixel color indicates cell $g_{K_{s}}$ value (same color scale as in A). The shaded area indicates the time range of snapshots in F. **C,** Average E cell firing frequencies plotted as cell position on the E cell lattice showing highest firing within the regions or ’hotspots’ of low $g_{K_{s}}$. **D,** Dominant rhythmic activity of individual E cells (dark blue = none, light blue = theta band, green = gamma band and yellow = mixed, both gamma and theta) displayed on E cell lattice illustrating that E cells with lower $g_{K_{s}}$ values showed stronger and more stable gamma activity while cells with moderate $g_{K_{s}}$ values exhibited stronger theta rhythm. **E,** Power spectrum analysis of E cell network firing. High power exhibited in both theta (5-12Hz) and gamma (40-60Hz) rhythm bands. **F,** Distribution of $g_{K_{s}}$ values for cells showing specific rhythmic activity illustrating correlation of activity type with $g_{Ks}$ value (same color code as in D). **G**, Snapshots of E cell firing rates from 4500–4850 ms during the simulation shown in B (shaded area). Cells inside the orange contour lines have $g_{K_{s}}$ values less than 0.6 mS/cm^2^.

To illustrate the emergence of theta-gamma coupled firing activity due to a spatially complex ACh landscape, we generated a spatially heterogeneous $g_{K_{s}}$ mapping by randomly assigning n = 9 center positions of low $g_{K_{s}}$ regions or ’hotspots’ of radius r = 4.2 (units in minimal distance between model cells, Fig 2A).

Cells within the $g_{K_{s}}$ ’hotspots’ exhibited gamma band firing activity that was intermittently interrupted as activity moved to other regions with low $g_{K_{s}}$ values, with network activity cycling periodically through the hotspots with theta band frequency. As illustrated in the raster plot in Fig 2B, this activity pattern resulted in subpopulations of E cells showing theta-band modulated gamma activity, and high power in both the theta and gamma frequency ranges in overall network activity (Fig 2E). Individual E cell’s firing frequencies were directly correlated with their $g_{K_{s}}$ values (Fig 2C, 2D and 2F). Namely, E cells near the centers of low $g_{K_{s}}$ regions (i.e. corresponding to highest concentrations of ACh) had the highest firing frequencies (Fig 2C) with higher power gamma band activity (Fig 2D and 2F), while E cells with moderate $g_{K_{s}}$ values fired in theta band ranges. Cells outside the $g_{K_{s}}$ hotspots showed little activity.

For comparison, in S2 Fig we show sample raster plots and analysis of network wide activity patterns and cell spiking frequencies for homogeneous $g_{K_{s}}$ spatial maps. In these simulations, we have kept $g_{K_{s}}$ at the same value for all the neurons and varied the value between 0 and 1.5 mS/cm^2^. We observed emergence of networkwide gamma band oscillations, and cellular spiking in that frequency, for low values of $g_{K_{s}}$, while systematic theta band oscillations were not present across the entire $g_{K_{s}}$ range.

### Characterization of gamma and theta band activity caused by a single peak *g*_*Ks*_ distribution

**Fig 3:**
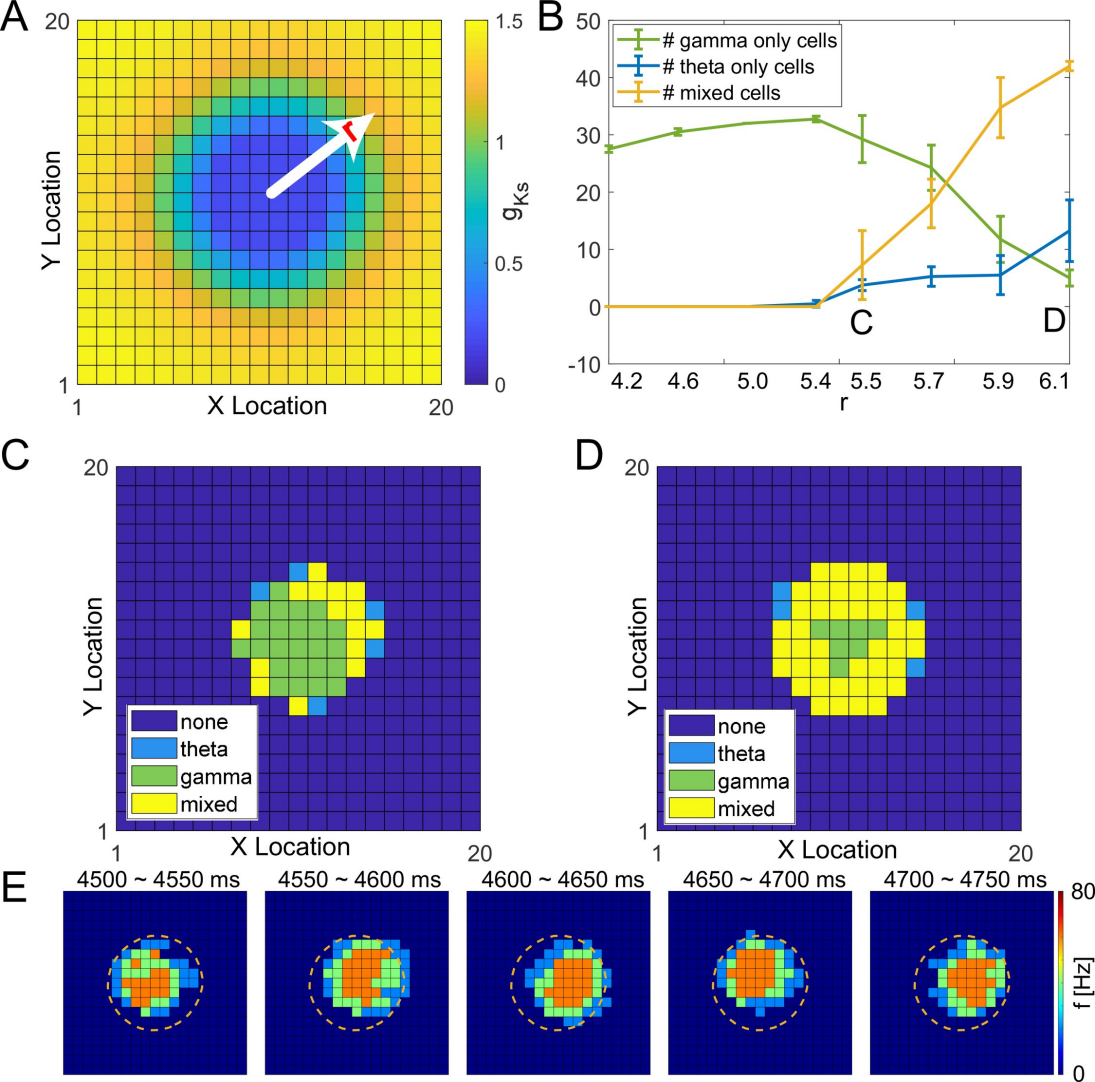
Gamma and theta band cell firing with a single peak g_Ks_ distribution. **A,** Example single peak *g*_*Ks*_ mapping projected on the 20×20 E cell lattice where r indicates the radius of the approximately circular region (hotspot) with low *g*_*Ks*_ values. **B,** The number of neurons primarily exhibiting gamma (green curve), theta (blue curve) and mixed rhythms (yellow curve) as a function of the radius of the *g*_*Ks*_ hotspot. **C, D,** Dominant rhythmic activity of individual E cells (dark blue = none, light blue = theta band, green = gamma band and yellow = mixed, both gamma and theta) plotted at cell position on the E cell lattice for *g*_*Ks*_ hotspot radius of *r* = 5.5 and *r* = 6.1, respectively. **E**, Snapshots of E cell firing activities from 4500–4750 ms during the simulation shown in D (*g*_*Ks*_ hotspot radius *r* = 6.1). Cells inside the orange contour lines have $g_{K_{s}}$ values less than 0.6 mS/cm^2^.

To understand how spatially heterogeneous distributions generate coupled theta-gamma activity, we next analyzed simple spatial *g*_*Ks*_ distributions. For a single low *g*_*Ks*_ region (Fig 3), firing activity was restricted to within the *g*_*Ks*_ hotspot by the higher neuronal excitability elicited by low *g*_*Ks*_ values and by the global inhibition provided to the rest of the network by the firing cells within the hotspot. Gamma range firing frequency of E cells within the *g*_*Ks*_ hotspot was generated by gating of E cell firing by local I cells through the PING mechanism [18–20]. As the radius r of the low *g*_*Ks*_ hotspot increased (without changing its minimum value; S3 Fig), the number of neurons exhibiting gamma slightly increased and theta band activity remained low (Fig 3B). As the hotspot radius increased past r = 5.5, however, the number of neurons primarily exhibiting gamma band activity started to decrease while the number of neurons firing at mixed theta and gamma ranges started to increase. While gamma neurons were located within the center of the *g*_*Ks*_ hotspot, theta neurons or neurons showing mixed theta-gamma band activity were located towards the hotspot’s outer edges (Fig 3C and 3D). Thus, theta band activity was generated due to firing activity spatially drifting within the *g*_*Ks*_ hotspot (Fig 3E).

This result echoed our previous findings [13] in similar 2D E-I networks with spatially uniform *g*_*Ks*_ values. In those networks, when *g*_*Ks*_ values were low, firing activity was spatially localized in a subregion of the network with surrounding cells showing intermittent firing, leading to drifting of an activity bump. When *g*_*Ks*_ values were higher (*g*_*Ks*_>0.2 mS/cm^2^), the bump exhibited translational motion across the whole network promoted by spike frequency adaptation mediated by the *g*_*Ks*_ regulated M-type K^+^ current. Here, similarly to those results, the movement of firing activity within *g*_*Ks*_ hotspots with larger radius resulted in theta band rhythmicity.

We further investigated how the emergence of theta/gamma oscillations due to a single *g*_*Ks*_ hotspot depended on the constant current applied to individual cells (which corresponds to external input) and the level of simulated ACh concentration (i.e. lower bound of *g*_*Ks*_ value within the hotspot, S4 Fig). The emergence of theta/gamma oscillations was very robust across these parameters. We generally observed that the oscillations emerged within the hotspot for lower minimal values of *g*_*Ks*_, except when neuronal external current was high and the oscillations were not confined to a hotspot but spread throughout the network. Another exception was for the lowest value of *g*_*Ks*_, when firing activity was stationary within the hotspot and only gamma oscillations were observed.

We additionally investigated how the theta and gamma oscillatory frequency changes with the size of the hot spot (S5 Fig). We observed that frequencies in both bands tend to decrease with the increase of hotspot radius. This is most likely due to the fact that as the hotspot increases in size the local feedback inhibition increases reducing the excitation individual cells receive.

Finally, we investigated how network topology (i.e. rewiring of the excitatory connectivity) affected the emergence of theta/gamma oscillations (S6 Fig). We observed that increasingly random E-E connectivity abolished theta band oscillations, while the presence of gamma band activity remained largely unchanged. This result suggests that the generation of theta-gamma coupling requires that synaptic excitation which is generally more local than synaptic inhibition in the network. We also observed a decrease in gamma frequency as a function of network rewiring. This is due to the fact that increasingly random connectivity promotes zero phase synchrony causing EPSPs mediated by spiking of presynaptic neurons to partially fall within the spike and/or refractory time of their postsynaptic targets, reducing their excitatory effect and the overall level of excitation in the network (S6B Fig).

### Characterization of theta/gamma band oscillations and their coupling emerging from a double peak g_Ks_ distribution

**Fig 4:**
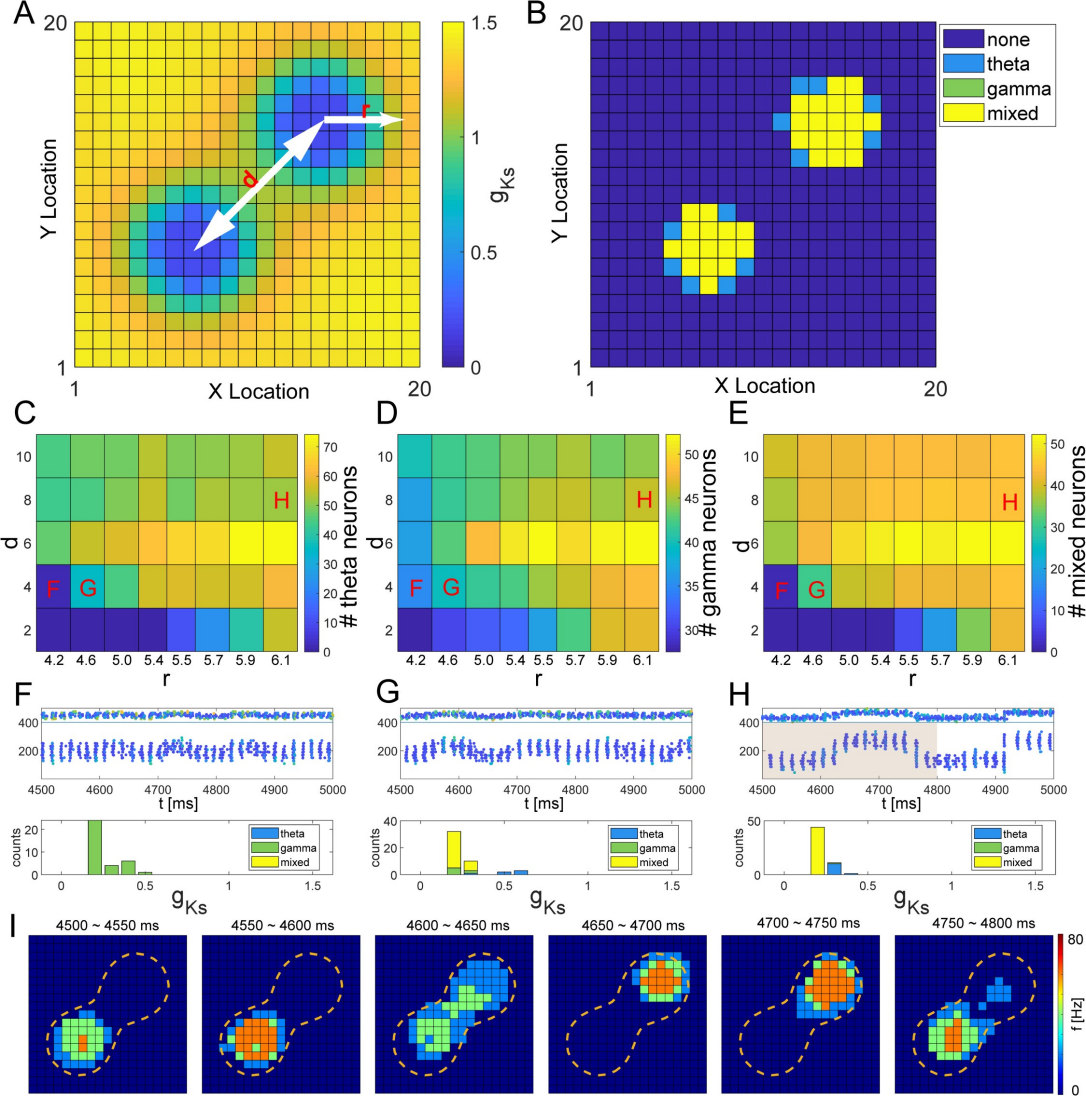
Theta/gamma band rhythms generated with a double peaked spatial distribution of g_Ks_. A, Example of double peaked spatial mapping of *g*_*Ks*_ values for corresponding neurons on the 20×20 E cell lattice for hotspot radius *r* = 4.6 and distance between hotspot centers *d* = 8. **B**, Dominant rhythmic activity of individual E cells (dark blue = none, light blue = theta band, green = gamma band and yellow = mixed, both gamma and theta) plotted at cell position on the E cell lattice due to double peaked *g*_*Ks*_ spatial distribution shown in **A**. **C, D and E**, Numbers of neurons exhibiting dominant theta (**C**), dominant gamma (**D**), or theta-gamma coupled activity (**E**) as a function of radius of *g*_*Ks*_ hotspots r and the distance between hotspot centers d for a double peaked *g*_*Ks*_ spatial distribution. **F, G and H**, Spike raster plots (top panels) and histograms for the dominant rhythmic activity exhibited by neurons based on their individual *g*_*Ks*_ values (bottom panels) for networks with double peaked *g*_*Ks*_ distributions with r and d values indicated by labels in C, D, E. In the raster plots, E cells are numbered 1 to 400, and I cells are numbered 401 to 500. Color indicates *g*_*Ks*_ values of cells with the scale in A. **I**, Snapshots of E cell firing rates from 4500–4800 ms during the simulation in H (shaded area in H; r and d values indicated by label H in C). Cells inside the orange contour lines have $g_{K_{s}}$ values less than 0.6 mS/cm^2^.

To investigate the emergence of rhythmic network activity when two spatially adjacent locations experienced cholinergic modulation at the same time, we considered the presence of two *g*_*Ks*_ hotspots in the network (Fig 4). Fig 4A shows an example of this kind of *g*_*Ks*_ mapping, in which *d* represents the distance between the two *g*_*Ks*_ hotspot centers while *r* denotes their radius (units in minimal distance between model cells). Here, cells located in the center of the hotspots exhibited theta-modulated gamma rhythms (mixed) while those on the peripheries of the hotspots showed primarily theta activity (Fig 4B). This occurred because spiking activity alternated between the hotspots at theta frequency. When a given hotspot was active it predominantly exhibited gamma band oscillation. This resulted in strong theta-gamma coupling as the gamma band oscillations appeared at a given site with theta band frequency (Fig 4B).

To analyze the properties of theta-gamma coupling as a function of the size and relative position of the hotspots, we varied the parameters *r* and *d* of the *g*_*Ks*_ spatial mapping and identified the numbers of cells showing primarily gamma or theta band activity, or theta-gamma coupled firing (Fig 4C, 4D and 4E). We observed that the numbers of neurons with theta, gamma and mixed rhythms increased monotonically with larger *r* (Fig 4C and 4E). At the same time, the highest number of cells representing all three types of dynamics (i.e. theta, gamma and mixed) happened around *d* = 6 with gamma and theta cell numbers declining faster for larger *d* than the mixed rhythm population. (Fig 4D).

To better understand these effects, we closely analyzed network dynamics for chosen sets of parameters. The parametric locations of the raster plots and cell statistics for different rhythms depicted on Fig 4F, 4G and 4H are indicated in Fig 4C, 4D and 4E. When *r* = 4.2 and *d* = 4 (Fig 4F), two *g*_*Ks*_ hotspots were small enough and sufficiently close to each other that cells in both spots fired simultaneously. Active neurons mainly fired with gamma rhythmicity except a few neurons with intermediate *g*_*Ks*_ values carried a theta rhythm. No neurons exhibited both rhythms in this case.

In contrast, for larger *r* and *d* values, cells in the two spots started to show alternating firing patterns (Fig 4G and even more so Fig 4H and 4I) with mixed theta-gamma rhythmicity. As described above, firing activity alternated between the two spots such that cells in each spot fired intermittently at gamma frequency with the alternation in activity between the spots occurring at theta frequencies. This alternation in firing between the hotspots occurred due to competition between the local excitation among cells within the hotspot, global inhibition generated by cells in the other hotspot and the magnitude of spike frequency adaptation (SFA) mediated by the activation of the M-current. Firing within the active hotspot mediated inhibitory signaling received by E cells in the silent hotspot. Due to their high excitability with low *g*_*Ks*_ values, feedback excitation with neighboring cells and small effects of SFA due to previously low activation, the silent hotspot E cells could start firing and inhibit the active hotspot. Subsequently as the SFA accumulated in the activated region the other hotspot can get triggered and take over. For larger radius values, theta-gamma activity dominated the network as activity moved consistently between the hotspots and only a few cells at the centers of the hotspots fired primarily at gamma frequency (Fig 4H).

All the simulation results presented here were performed for an M-current activation time constant of *τ*_*z*_ = 75*ms* following [21]. To better understand how the M-current timescale affects frequency of theta and gamma band oscillations, we performed additional simulations scanning different values of *τ*_*z*_∈[25*ms*, 125*ms*] (S7 Fig). As expected, we observe a significant slowdown of theta band frequency with increased *τ*_*z*_, while no systematic changes were detected in gamma band oscillations.

We also analyzed how theta and gamma frequency changed as a function of *g*_*Ks*_ hotspot size and the distance between hotspot centers (S8 Fig) and as a function of connectivity of the excitatory subnetwork (S9 Fig). We observed that frequencies of both theta and gamma oscillations decreased as the hotspots’ radius increased. However, the frequencies of the two oscillations showed opposite trends with the increase of hotspot center distance–frequency of the theta band decreased while the gamma frequency generally increased. We attribute this effect to the fact that as the two hotspots are positioned farther apart it takes longer for them to switch their activations. When excitatory connectivity was made increasingly random (S9 Fig), activity switching between the two hotspots stopped and firing became synchronous with cells in both hotspots exhibiting gamma band oscillations.

Finally, we investigated whether similar dynamical switching between the hotspots would occur if, instead of *g*_*Ks*_ hotspots, increased excitability was driven with additional external current in two hotspots ($I_{drive}^{i}$; see Materials and Methods), with a homogeneous *g*_*Ks*_ map across the network (S10 Fig). We observed that only for very narrow range of g_Ks_ values is it possible to get localized, selective activity switching between the two stimulation hotspots with theta frequency (namely for *g*_*Ks*_ ~0.2). For other values of *g*_*Ks*_, activity switching does not take place, or is not specific to the neurons within the hotspots (i.e other neurons outside the hotspots become activated; S10 Fig–black colored spikes in addition to red spikes). This non-specific activation obliterated theta band oscillations. Thus, we interpret these results that while emergence of theta/gamma oscillations may be possible in response to heterogeneous patterns of external drive, they occur for a narrow band of network *g*_*Ks*_ modulation and they are significantly less robust.

### Variability of theta/gamma band oscillations with spatially random *g*_*Ks*_ distributions

The analysis in the preceding sections illustrates that, in networks with local excitation and global inhibition, theta-modulated gamma band firing occurred in cells on the outer edges of individual *g*_*Ks*_ hotspots or, if the *g*_*Ks*_ hotspot was large compared to the excitation range, within the hotspot itself. With multiple, spatially separated *g*_*Ks*_ hotspots, all cells within *g*_*Ks*_ hotspots can exhibit theta-modulated gamma band firing as competition between local excitation and global inhibition within and between hotspot cells causes alternation of firing episodes. These results indicate that both sizes as well as relative positions of the hotspots matter. In the more biologically realistic scenario of spatially random *g*_*Ks*_ distributions consisting of multiple hotspots, these same mechanisms contribute to generating theta-gamma coupled firing, however we found that resulting strengths of theta and gamma band activity were highly variable, depending on the specific realization of the *g*_*Ks*_ spatial map (i.e. positions of the hotspots).

**Fig 5:**
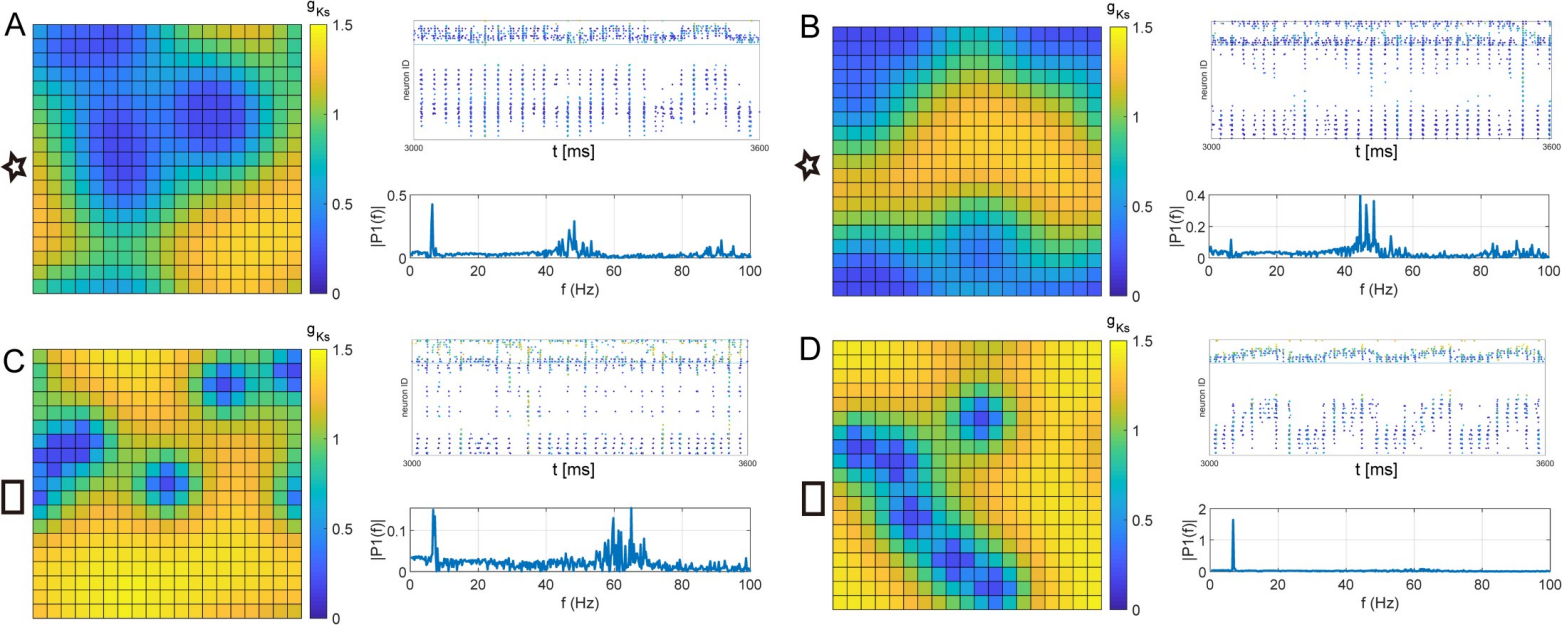
Examples of variability in theta/gamma rhythms for randomly generated spatial *g*_*Ks*_ distributions. The *g*_*Ks*_ spatial mappings (color plots), network spike raster plots (right panels, top) and network frequency power spectrum (right panels, bottom) for two pairs (**A, B** and **C, D**) of *g*_*Ks*_ map realizations with the same sets of parameters. **A, B** The results of 2 random *g*_*Ks*_ mappings with 6 hotspots of radius r = 5.4 (marked by ’star’ in S5 Fig). **C, D** The results of 2 random *g*_*Ks*_ mappings with 6 hotspots of radius r = 2.8 (marked by ’square’ in S5 Fig).

As an example, Fig 5 shows different realizations of *g*_*Ks*_ spatial mappings with 6 hotspots of radius *r* = 5.4 (Fig 5A and 5B, star in S5 Fig) and *r* = 2.8 (Fig 5C and 5D, square in S8 Fig). Despite the same hotspot properties, the number and size of effective low *g*_*Ks*_ regions was varied as hotspots could overlap and coalesce if their centers were next to each other. Network dynamics were likewise highly variable with some mappings showing clear coexistence of theta/gamma rhythms (Fig 5A and 5C) while in others gamma (Fig 5B) or theta (Fig 5D) power dominated. This is due to the fact that for the *g*_*Ks*_ mappings with coexisting theta/gamma rhythms, individual hotspots coalesced into larger but spatially distinct low *g*_*Ks*_ regions (Fig 5A and 5C; note that networks have periodic boundary conditions so hotspots may wrap around the lattice). In these cases, theta-gamma coupling was generated similarly as in the double peaked *g*_*Ks*_ spatial distribution. On the other hand, gamma power dominated when the individual *g*_*Ks*_ hotspots coalesced into an effective single low *g*_*Ks*_ region (wrapped around the corners of the network, Fig 5B). The strongest theta power was generated when hotspot centers were more evenly dispersed across the network and activity traveled successively along the low *g*_*Ks*_ regions (Fig 5D). This observed variability in theta/gamma power provides a possible insight into experimental inter-animal variability, as the relative locations of ACh release sites could be highly individualized within the experimental subjects.

To gain a better understanding of the variability in observed network rhythmicity, we simulated effects of random *g*_*Ks*_ mappings having different numbers of hotspots of different sizes (S11 Fig) and measured mean theta and gamma power (and their ratio) and their relative standard error (RSE) across simulation runs with different instantiations of the mappings. We additionally measured peak theta and gamma frequency for each of these hotspot distributions (S12 Fig). As shown in the example above, the relative power amplitudes varied significantly for maps consisting of the same number of hotspots having the same sizes, reporting relatively high RSE, except for the situation when only one hotspot was present.

### Vicinity to high ACh region modulates strength of theta/gamma coupling

**Fig 6:**
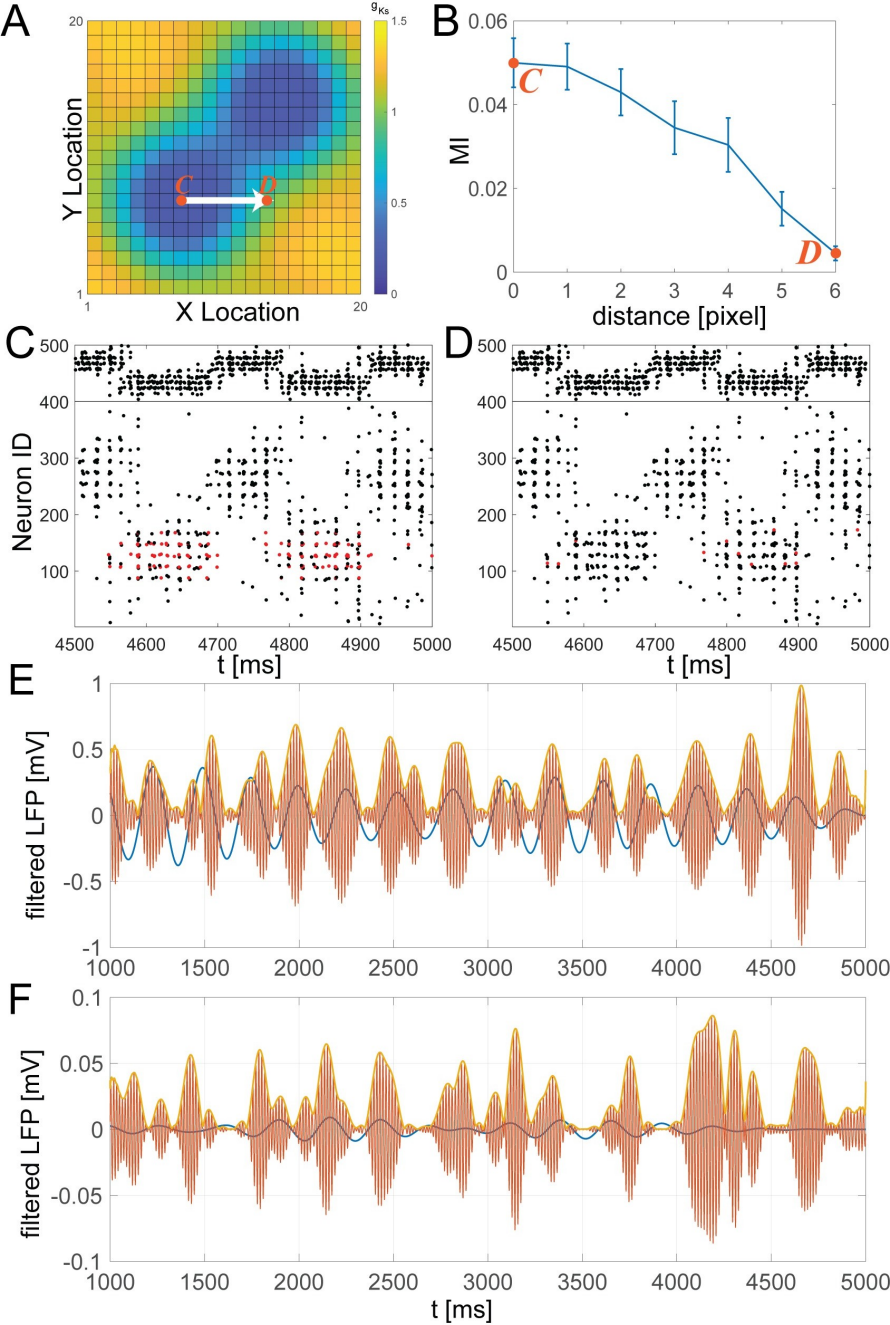
Strength of theta-gamma coupling as a function of the distance from the low *g*_*Ks*_ region. A local field potential (LFP) signal was constructed from spike trains of E cells at different distances from the center of a *g*_*Ks*_ hotspot. **A**, Double peaked *g*_*Ks*_ spatial mapping with locations marked to calculate LFPs. **B,** Modulation Index (MI) between gamma and theta filtered LFP traces as a function of the distance from the center of the *g*_*Ks*_ hotspot (as indicated in A.) **C and D,** Example raster plot of the network-wide spiking near the hotspot (C, as marked on A) and away from the hotspot (D, as marked on A). E cells are numbered 1 to 400, and I cells are numbered 401 to 500. Cells used to compute the LFP signal are marked in red. **E and F,** Examples of theta (blue curve) and gamma (orange curve) filtered LFP signals computed at locations near (C) and far (D) from the hotspot, marked on A, B.

It has been shown experimentally that ACh release promotes theta-gamma coupling, and furthermore, this coupling is specifically mediated via M1 muscarinic receptors [8,15]. In our simulations, cells showing the strongest theta-gamma coupling were located within *g*_*Ks*_ hotspots, when a single large hotspot (Fig 3), or more than one hotspot were present (Fig 4). To investigate how the strength of theta-gamma coupling varied with location relative to the position of the hotspots, we constructed local field potential (LFP) signals at different distances from a *g*_*Ks*_ hotspot (Fig 6). Here, specifically, we used a double peaked *g*_*Ks*_ spatial mapping (Fig 6A) since this network showed the most robust theta/gamma coupling. The locations at which LFP was calculated is bounded by “C” and “D”, corresponding to the respective panels showing rasterplots from those locations (Fig 6C and 6D, red dots correspond to spikes of the neurons used for LFP calculation at these respective locations). The LFP signal was computed from the spike trains of the 12 E cells closest to the marked location (see Materials and Methods). For completeness, S13 Fig shows the results for an LFP signal generated directly from the voltage traces of the selected cells, with results remaining qualitatively the same. The locations were chosen to be progressively further away from the center of one of the *g*_*Ks*_ hotspots (x-axis in Fig 6B). Separately filtering the LFP signal in the theta and gamma bands (Fig 6E corresponding to location C and Fig 6F corresponding to location D), we calculated the modulation index (MI) to quantify the coupling strength between the two signals as described in the Materials and Methods section. MI values as a function of distance from the *g*_*Ks*_ hotspot center decreased (Fig 6B), showing that theta-gamma coupling strength decreased with distance from the *g*_*Ks*_ hotspot.

Gamma frequency spiking is evident primarily at the locations which have low *g*_*Ks*_ (high ACh; In Fig 6C, blue dots correspond to spikes of neurons located within the *g*_*Ks*_ hotspot as color coded on Fig 6A). This is due to the fact that only these locations can generate reliable PING dynamics. Away from these regions, even if activity of the network briefly traverses a given location, the gamma oscillations will be greatly reduced or not present at all (due to lack of excitability mediated by low *g*_*Ks*_). Thus, the diminished theta-gamma coupling away from the *g*_*Ks*_ hotspot is due to reduction of local gamma oscillations. Examples of the two filtered LFP signals at different network locations are shown in Fig 6D and 6E.

### Effect of *g*_*Ks*_ modulation on network response to external stimuli

Behavioral attentional tasks, particularly visual attention tasks, report differences in responses to sensory-mediated stimuli depending on whether such stimuli undergo attentional processing [22,23]. Localized cholinergic signaling was shown to be necessary and sufficient for the attentional elevation of the processing of stimuli [24,25].

**Fig 7:**
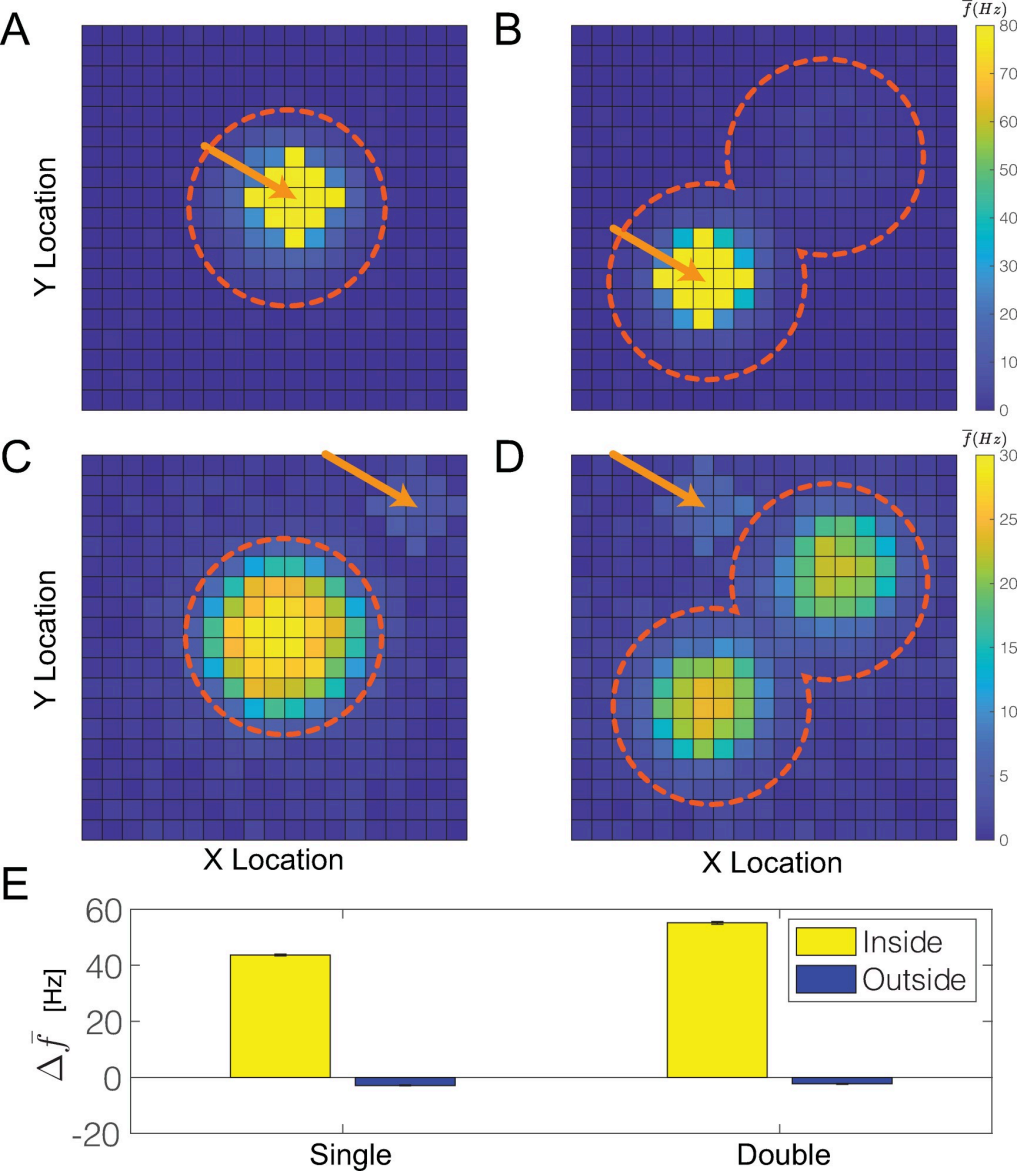
Firing response to an external stimulus with single and double peak *g*_*Ks*_ spatial distributions. **A, B, C, D,** Average E cell firing frequencies plotted as cell position on the E cell lattice showing varied firing responses to an external excitatory stimulus targeting a subset of E cells. Dashed circles indicate location and radius of ***g***_***Ks***_ hotspots and arrows indicate location of targeted cells. Targeted cells were either inside (A, B) or outside (C, D) of the ***g***_***Ks***_ hotspot. **E,** The change in average firing responses of the targeted E cells to the external stimulus relative to their responses in the absence of ***g***_***Ks***_ modulation (homogeneous ***g***_***Ks***_ at default value). Yellow (blue) bars correspond to when targeted E cells are inside (outside) the ***g***_***Ks***_ hotspot. The four bars from left to right correspond to cases shown in A, C, B and D, respectively.

To investigate how spatially heterogeneous *g*_*Ks*_ modulation (simulating ACh mediated attentional drive) affects responses to external (sensory) stimuli, we measured relative changes in firing frequency of a subset of excitatory cells targeted by an external excitatory drive, $I_{drive}^{i}$, when the targeted E cells were inside or outside of a *g*_*Ks*_ hotspot (i.e. within or outside attentional drive, respectively). We compared three situations corresponding to three behavioral conditions: 1) targeted E cells are inside the *g*_*Ks*_ hotspot corresponding to presentation of a sensory stimulus which is attended to; 2) targeted E cells are outside the *g*_*Ks*_ hotspot corresponding to presentation of a sensory stimulus but attention is directed elsewhere; and finally 3) the subset of E cells is targeted by the external drive but there is no *g*_*Ks*_ modulation in the network (spatially homogeneous *g*_*Ks*_ at the default value) corresponding to presentation of a sensory stimulus in the absence of attention. These conditions were simulated for single (Fig 7; left column) and double (Fig 7; right column) peaked *g*_*Ks*_ spatial mappings.

We observed that for the spatial mapping with single peak *g*_*Ks*_, the firing response of the targeted neurons (Fig 7A; location marked by arrow) was significantly higher when they were located in the *g*_*Ks*_ hotspot (condition 1), relative to their response to the stimulus in the absence of *g*_*Ks*_ modulation (condition 3) (Fig 7A). When targeted cells were outside the *g*_*Ks*_ hotspot (Fig 7C location marked by arrow), their firing response to the stimulus (condition 2) was significantly suppressed compared to their response in the absence of *g*_*Ks*_ modulation (condition 3) (Fig 7C and 7E). This suppression was due to the global inhibition induced by the cells firing in the *g*_*Ks*_ hotspot that attenuated the response to the external stimulus.

For the spatial mapping having two *g*_*Ks*_ peaks, responses of targeted cells were generally the same as for the single hotspot case whether the targeted cells were located inside or outside of a *g*_*Ks*_ hotspot (Fig 7B and 7D). We additionally observed that when the targeted cells were in one of the *g*_*Ks*_ hotspots, their firing dominated the network, shutting down activation at the other hotspot. This led to abolition of theta-gamma coupling as the neurons in the targeted hotspot fired at gamma frequency.

## Discussion

Cholinergic signaling is necessary and sufficient for the detection of cues in attentional contexts. Moreover, cholinergic signaling influences the degree of forebrain desynchronicity across circadian stages. Until recently it has been thought that cholinergic signaling occurs at a relatively low temporal resolution but also with highly limited spatial selectivity. In contrast, recent results indicated that ACh release is more localized and asynchronous within activated brain modalities (for example, Fig 1). Such evidence for spatially heterogenous ACh signaling has remained rare, in part because until the advent of biosensors and, more recently, the GRAB_ACh_ sensor, prior methods available for monitoring ACh release did not allow for measurements at a relevant spatial resolution. However, using the ACh3.0 GRAB sensor, spatially heterogenous cholinergic signaling was recently shown in the somatosensory cortex of walking mice. Cholinergic hotspots with a diameter of about 40 μm were selectively activated by runs and appeared to be surrounded by inactive areas of similar diameters (see Fig 3O in [26]). Using a prior version of this sensor to monitor ACh release in hippocampal slices, the size of hotspots was concluded to be even smaller, about 16.5 μm in diameter [27]. The aim of this modeling study was to understand how a highly heterogenous distribution of ACh affects rhythmic network firing activity.

We have previously shown [12], using biophysical computational modeling, that a network of randomly coupled excitatory and inhibitory neurons can generate transient gamma oscillatory activity in response to simulated spatially global but temporally brief pulses of ACh. This effect was mediated by blockage of the M-type K^+^ current (i.e. *g*_*Ks*_ conductance). Depending on network connectivity, gamma activity decayed with the simulated *g*_*Ks*_ transient modulation or was sustained in the network after the *g*_*Ks*_ transient completely dissipated.

In contrast, the present study demonstrates that in a network with local excitation/global inhibition connectivity, spatially heterogenous ACh modulation, modeled by varying the M-current differently in different cells, leads to the emergence of spatially localized theta and gamma band activity rhythms.

The appearance of the individual rhythms and theta-modulated gamma oscillations strongly depended on the specific features of the spatial distribution of the maximal conductance of the M-current, *g*_*Ks*_ (i.e. the number of *g*_*Ks*_ hotspots and their radius). Furthermore, we identified two basic mechanisms mediating strong theta-gamma coupling. First, if a single *g*_*Ks*_ hotpot is present and its size is larger spatially than the scope of the local excitation (i.e. range of E-E connectivity), the neuronal activation moves throughout the population encompassed by the hotspot. Second, when two or more *g*_*Ks*_ hotspots are present, the neuronal activity alternates between the hotspots. In both cases, the activated population exhibits gamma band activity. The emerging gamma rhythm is mediated via the PING mechanism, whereas the emerging theta rhythm is caused by activity traversing *g*_*Ks*_ hotspots, mediated by SFA.

Together, these simulations revealed that spatially heterogenous release of ACh leads to the manifestation of theta and gamma oscillations and their coupling. For spatially homogeneous *g*_*Ks*_ distributions, when *g*_*Ks*_ is low there is effectively no spike frequency adaptation in the network and thus gamma oscillations may be supported indefinitely (S2 Fig). On the other hand, homogeneous *g*_*Ks*_ distributions at high values lead to random neuronal spike-frequency adaptation patterns, without consistent formation of theta band oscillations. However, spatially localized and constrained regions of low *g*_*Ks*_ allow for theta band modulation of the activity within these regions with gamma oscillations emerging during phases of firing activity. Furthermore, the specific pattern and frequencies of observed oscillatory activity is highly dependent on the specific distribution of ACh release sites and neuronal network structure. For example, the specific frequencies observed in the theta and gamma range depend on network wiring topology and the cellular signaling properties (i.e. excitatory cell input or inhibitory time constant; S14 Fig, supplemental material). These *in silico* network dynamics reproduce important *in vivo* results, as discussed below.

Network connectivity plays a role in supporting the emergence of theta-band activity. The local excitation/global inhibition connectivity structure allows for firing activity in discrete and localized network sites, with the *g*_*Ks*_ distribution controlling the location and sequence of activated sites. We have shown, that similar results are obtained when inhibitory neurons had sparser random connectivity to both excitatory and inhibitory targets (S15 Fig, supplemental material). In general, as long as inhibition has a larger footprint than excitation in the network, qualitatively similar dynamics will be obtained. Such connectivity is thought to be found on the meso-scale in the connectivity of functional modules [28]. In addition we believe that a larger inhibitory footprint could be potentially obtained via long range excitatory connections that specifically target locally connected inhibitory cells.

Brain rhythms in the theta (∼ 5 − 12Hz) and gamma (∼ 30 − 100Hz) frequency bands have been shown to critically contribute to essential cognitive processes in many brain regions, including the cortex and hippocampus [19,20,29–32]. Recently, the coupling of these rhythms, such that the amplitude of gamma band activity is modulated by the phase of theta band activity, has been identified as a key component of the neuronal local field potential (LFP) or electrocorticogram (ECoG) observed during perceptual, attentional and other cognitive processes [22,33–37]. Such cross-frequency coupling has been proposed to signify the cooperation of diverse circuits and neuronal populations in order to integrate multiple cognitive operations and force the execution of stimuli-bound cognitive or behavioral outputs [38,39]. For example, in rats performing a signal-detection based attention task, successful performance of the task was shown to be dependent on the appearance of theta-modulated gamma band activity in the LFP measured in the prefrontal cortex (PFC) [15]. Generation of coupled theta-gamma activity was associated with fast (or transient), cue-bound cholinergic signaling in the PFC. Importantly, disruption of post-synaptic ACh signaling, by blocking muscarinic M1 acetylcholine receptors (mAChRs), attenuated gamma synchronicity, disrupted theta-gamma coupling and caused detection failures [15]. Studies in hippocampal and entorhinal cortical circuits have similarly shown that ACh signaling promotes theta-gamma coupling [16]. Our simulation results suggest that such muscarinic-dependent theta and gamma activity may be generated by spatially heterogeneous modulation of neural properties due to spatially circumscribed release of ACh.

The above cited results concentrate on theta and gamma oscillatory components found in the LFP signal, rather than directly in neural spiking activity. There are a limited number of studies suggesting that local increases in ACh signaling can lead to increases in gamma periodic multiunit spiking in the neocortex [40] and theta periodic spiking in the hippocampus [41]. However, to our knowledge the relationship of ACh and theta-gamma LFP coupling to spiking of neurons has not been studied *in vivo*. Clearly, there is a substantial need for such research.

Previous computational models for theta-gamma coupling activity in E-I networks relied on the presence of two populations of inhibitory cells that gate the firing of E cells with different time scales [42–46]. Namely, a fast I population generates E cell firing at gamma frequencies through the PING mechanism, while a slower I population gates the gamma oscillatory firing at theta frequencies. In the novel mechanism we describe here, obtaining theta band rhythmicity is mediated by activation of muscarinic receptors that modulate activity on timescales corresponding to theta band oscillation via SFA. Here, both excitatory and inhibitory cell populations are endowed with the M-current, however similar results are observed when muscarinic receptors are expressed (within the model) on excitatory pyramidal cells only.

In our networks, the strength of theta-gamma coupling varied with distance from the center of a *g*_*Ks*_ hotspot. In LFP measurements within the hotspot, gamma oscillations were tightly aligned with the peaks of the theta rhythm. This functional coupling decreased for locations away from the hotspot, leaving the theta and gamma band activity largely uncoupled. Although the available data from *in vivo* recordings already support the view that theta-gamma coupling is caused by, and occurs in the region of, elevated cholinergic signaling and muscarinic M1 receptor stimulation [15], prior electrochemical recording techniques have not achieved levels of spatial resolution that would allow the characterization of hotspots and their "colder" boundaries. More recent G-protein coupled ACh sensors appear capable of differentiating levels of cholinergic signaling on a scale of tens of micrometers [27]. However, measuring LFPs simultaneously with fluorescence imaging remains challenging and, for the observation of theta-gamma coupling, may require simultaneous recordings during task performance [15]. Progress in the development of *in vivo* recording techniques may soon allow a direct test of the neurobiological validity of our findings.

We observed that spatially localized *g*_*Ks*_ hotspots acted to gate responses to external excitatory input to the network. External stimuli applied to E cells within a hotspot generated a dramatic increase in spiking frequency of stimulated neurons and, to a smaller degree, around the network, as compared to the response of the stimulus in the absence of *g*_*Ks*_ modulation. Conversely, when external stimuli were applied to cells located outside the *g*_*Ks*_ hotspot, stimulated cells showed a significantly reduced response compared to their response in the absence of *g*_*Ks*_ modulation. These results mirror electrophysiological findings from primate visual cortex, where attention localized to neurons’ retinotopic field augment firing rate responses [47] as well as coherence of firing with theta and gamma oscillations [22,37]. This effect provides a mechanism not only to aid in detection and discrimination of sensory stimuli in attended locations [39], but also may aid in selective communication of responses to attended stimuli between distant cortical areas [22,37].

Our present results suggest that cholinergic activity influences target circuitry in a highly spatially heterogenous manner which influences the locations of cellular rhythmic activity. Results presented here consider “time-frozen” *g*_*Ks*_ (ACh) spatial distributions while in reality, they will be continually changing. The time scale of ACh diffusion/uptake can be inferred from Fig 1C to be somewhere between 5-15s, which is clearly long enough to establish stable theta/gamma band rhythms by the presented mechanism. Further development of our model by integrating the impact of such temporal dynamics, may have interesting implications for understanding the neuronal mechanisms contributing to, for example, the ‘Biased Competition’ model of attention, that is, the mechanisms that allow behaviorally significant stimuli to undergo feature extraction, while the processing of competing stimuli, even if placed in the same receptive field, is suppressed [48,49].

With our prior neurobiological and computational findings about the functional significance of fast, transient cholinergic signals, conceptualizations about the regulation and function of cholinergic neurons have advanced from traditional views about monolithic neuromodulator actions to temporally and spatially differentiated signaling across, for example, cortical layers and microcolumns. Together with our present results, this new paradigm for cholinergic signaling suggests novel underlying mechanisms for how cholinergic activity can rapidly re-direct information flow in target circuitry and thus play an essential role for maintaining behavioral and cognitive flexibility [8,50].

## Materials and methods

### Experimental recordings

The evidence depicted in Fig 1 was adopted from experiments which measured fast, transient cholinergic signals (“transients”) across circadian cycles in the cortex and hippocampus [51]. Data recorded in prelimbic cortex are shown here. The four platinum (Pt) recording sites were fabricated onto a ceramic backbone electrode where the upper and lower pairs of recording sites were separated by 100 μm. The data shown were recorded via an upper sensor (“sensor 1) and a lower sensor (“sensor 2”). The neurochemical recording scheme, was previously described in detail, while amperometric measures were validated in terms of reflecting newly released acetylcholine (ACh) [10,52–54]. Briefly, newly released ACh is hydrolyzed by endogenous acetylcholinesterase (AChE), and the resulting choline is oxidized by choline oxidase immobilized onto the Pt electrodes. The resulting hydrogen peroxide is oxidized electrochemically and current yields are recorded amperometrically. m-phenylenediamine (mPD) was electropolymerized onto the electrodes to enhance the selectivity for detecting analyte relative to currents produced by potential electroactive interference. Electrodes were calibrated prior to implantation into the brain. Electrochemistry data were collected via the FAST-16 recording system at a sampling frequency of 20 Hz. Electrophysiological signals were acquired at 128 Hz for sleep scoring analysis as previously reported (Opp ICELUS Acquisition program: [55]. Scored sleep states include rapid eye movement (REM) sleep, slow-wave sleep (SWS) and waking (WAKE) periods.

### Cortical neuron model

Neuron membrane potential dynamics were described by a Hodgkin-Huxley based model of cholinergic modulation in pyramidal cells [21,56]. The effects of ACh as mediated through muscarinic ACh receptors were shown to be well modeled by varying the maximum conductance *g*_*Ks*_ of a slow, low-threshold K^+^ mediated adaptation current. The model also featured a fast, inward Na^+^ current, a delayed rectifier K^+^ current and a leakage current. With *C* = 1*μF* /cm^2^, units of V_i_ being millivolts and units of t being milliseconds, the current balance equation for the *i*^*th*^ cell was

$$C\frac{dV_{i}}{dt}=-g_{Na}m_{i,\infty }^{3}h_{i}(V_{i}-V_{Na})-g_{K_{dr}}n_{i}^{4}(V_{i}-V_{K})-g_{K_{s}}^{i}z_{i}(V_{i}-V_{K})-g_{L}(V_{i}-V_{L})+I_{drive}^{i}-I_{syn}^{i}+I_{noise}^{i}$$

where a constant current $I_{drive}^{i}$ was externally applied and $I_{syn}^{i}$ represented the synaptic current received by the *i*^*th*^ neuron.

For some simulations we added noisy input current pulses $I_{noise}^{i}$ dictated by a Poisson process (Poisson Rate $\lambda =\frac{1}{150}{\mathrm{ms}}^{-1}$) with amplitude of 6*μA*/*cm*^2^ and duration 1ms.

Activation of the inward *Na*^+^ current was instantaneous and governed by the steady-state activation function *m*_*i*,∞_(*V*_*i*_) = {1+exp[(−*V*_*i*_−30.0)/9.5]}^−1^. The *Na*^+^ inactivation gating variable *h*_*i*_ was governed by

$$\frac{dh_{i}}{dt}=\frac{h_{\infty }(V_{i})-h_{i}}{\tau_{h}(V_{i})}$$

where *h*_∞_(*V*) = {1+exp[(*V*+40.5)/6.0]}^−1^ and *τ*_*h*_(*V*) = 0.37+2.78{1+exp[(*V*+40.5)/6.0]}^−1^.

The delayed rectifier *K*^+^ current was gated by *n*_*i*_, the dynamics of which was given by

$$\frac{dn_{i}}{dt}=\frac{n_{\infty }(V_{i})-n_{i}}{\tau_{n}(V_{i})}$$

where *n*_∞_(*V*) = {1+exp[(−*V*−30.0)/10.0]^−1^ and *τ*_*n*_(*V*) = 0.37+1.85{1+exp[(*V*+27.0)/15.0]}^−1^.

**Fig 8:**
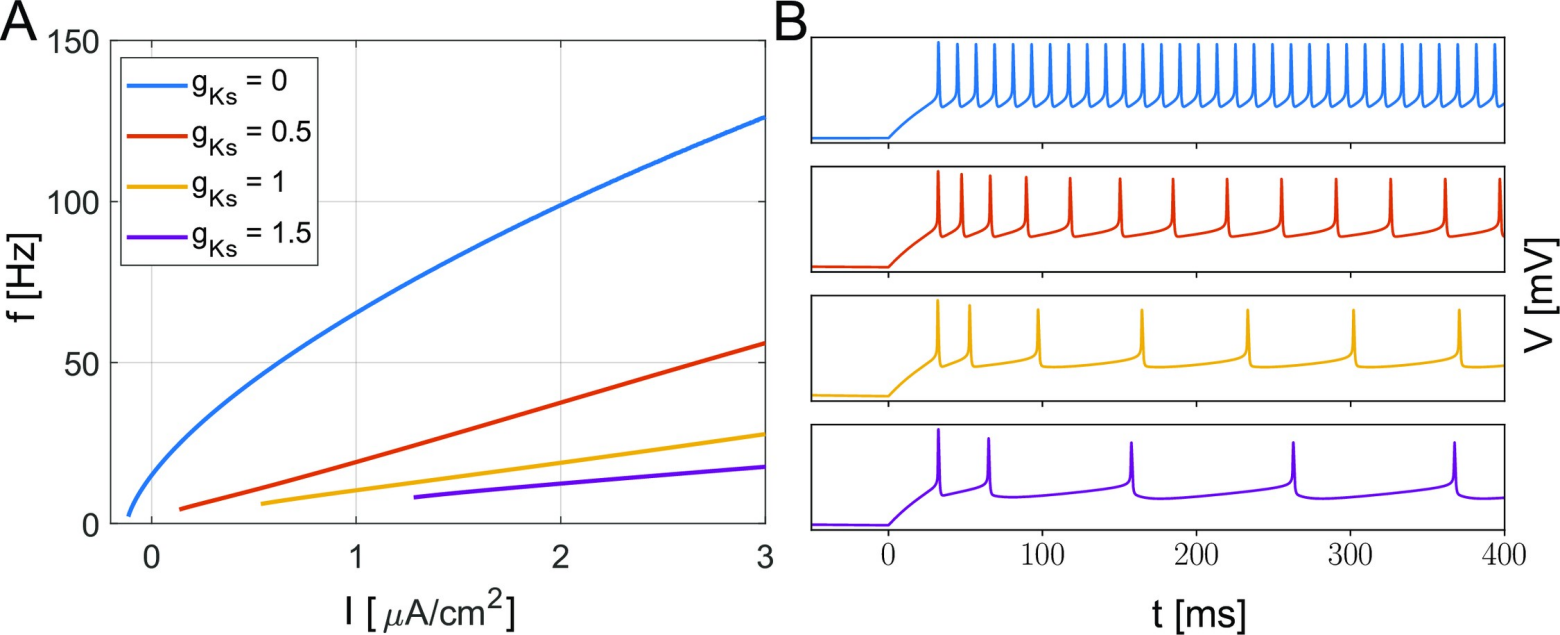
Cholinergic modulation of neuron frequency-current relationship. **A**, Frequency-current (f-I) curve of the cortical neuron model with different *g*_*Ks*_ values simulating different levels of ACh neuromodulation (*g*_*Ks*_ = 0 mS/cm^2^ for high ACh modulation and *g*_*Ks*_ = 1.5 mS/cm^2^ for no ACh neuromodulation). **B**, Spike-Frequency-Adaptation (SFA) showed by the voltage traces of different *g*_*Ks*_ values with current of 1.5μA/cm^2^ being applied (Same color code with **A**).

To model ACh blockade of the muscarine-sensitive M-current observed in cortical neurons, the maximum conductance of the slow, low-threshold *K*^+^ current in the *i*^*th*^ cell, $g_{K_{s}}^{i}$, was varied between 1.5 mS/cm^2^ for no ACh modulation and 0 mS/cm^2^ for strong ACh modulation. In this model neuron, decreasing values of *g*_*Ks*_ increase membrane excitability as reflected in the frequency-current relation (Fig 8), as well as affect spike-frequency adaptation and the neural phase response curve [56,57]. The dynamics of the corresponding gating variable *z*_*i*_ were governed by

$$\frac{dz_{i}}{dt}=\frac{z_{\infty }(V_{i})-z_{i}}{\tau_{z}}$$

where *z*_∞_(*V*) = {1+exp[(−*V*−39.0)/5.0]}^−1^. These M-current kinetics are similar to M-current models in [58] and [59]. Values of other parameters were: *g*_*Na*_ = 24.0*mS*/*cm*^2^, $g_{K_{dr}}=3.0mS/cm^{2}$, *g*_*L*_ = 0.02*mS*/*cm*^2^, *V*_*Na*_ = 55.0*mV*, *V*_*K*_ = −90.0*mV* and *V*_*L*_ = −60.0*mV*, *τ*_*z*_ = 75*ms*.

### Network model

**Fig 9:**
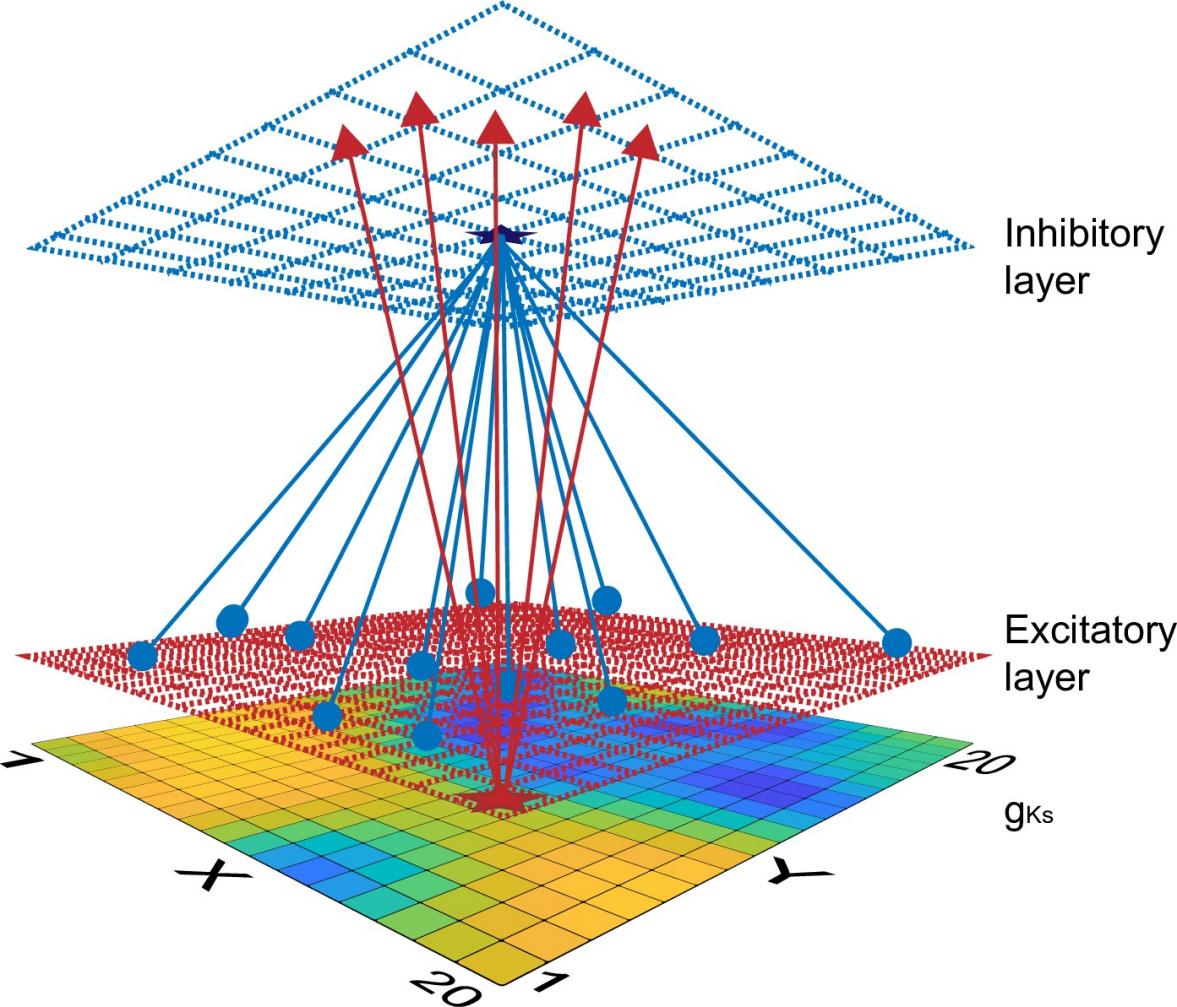
Schematic showing E—I network connectivity and spatially heterogeneous $g_{K_{s}}$ distribution. Network consisted of 100 inhibitory neurons (blue top layer) and 400 excitatory neurons (red middle layer) evenly distributed over square lattices periodic boundary conditions. E-E and E-I synapses were short range (red arrows) while I-I and I-E synapses (blue arrows) were global. Bottom layer shows an example of a heterogeneous $g_{K_{s}}$ spatial distribution mapping (yellow to blue indicates high to low $g_{K_{s}}$ values).

We simulated two-dimensional networks with 400 excitatory (E) neurons and 100 inhibitory (I) neurons evenly distributed over separate square lattices (20×20 E cell lattice and 10×10 I cell lattice, Fig 9). The inhibitory cells accounted for 20% of cells similar to what has been reported experimentally in the cortex [60]. A local excitation-global inhibition network topology (similar to center-surround or lateral inhibition topologies) was used in which E cells sent outgoing connections to their 40 nearest neighbors on the E cell lattice and to their 10 nearest neighbors on the I cell lattice. Inhibitory cells sent outgoing connections to all E cells and all I cells. Periodic boundary conditions were imposed on cells near the lattice edges. This is an established network scheme for cortical connectivity with the ability to balance short-range excitation and global inhibition [61].

To investigate how the observed results persist with random but sparse inhibitory connectivity, we performed additional simulations where we reduced the density of inhibitory connectivity to as low as 40% (S15 Fig). The observed network dynamics did not change qualitatively.

To illustrate network dynamics on a raster plot, we indexed neurons by lattice column such that a neuron’s index. *ID*_*i*_, was set to the sum of its lattice y-coordinate and the product of its lattice x-coordinate with the length of lattice network, *ID*_*i*_ = *y*_*i*_+*x*_*i*_×*L* (*L* =20 for E-cells and *L* = 10 for I-cells). The first 400 indices were assigned to E-cells while I-cells’ indices ranged from 401 to 500.

The synaptic current $I_{syn}^{i}$ represented the total synaptic current received by neuron *i* and was given by $I_{syn}^{i}=\sum_{j}I_{syn}^{ij}$ where $I_{syn}^{ij}=w_{ij}\sum_{k}\exp\left(-\frac{t-t_{jk}}{\tau }\right)(V_{i}-E_{syn}^{j})$ at times *t*>*t*_*jk*_ (spike time of *j*^*th*^ neuron’s *k*^*th*^ spike). *w*_*ij*_ is the *ij*^*th*^ element in the adjacency matrix for the weighted directed graph for synaptic connections in our network model. We used 0.01 mS/cm^2^, 0.05 mS/cm^2^, 0.04 mS/cm^2^ and 0.04 mS/cm^2^ for E-E, E-I, I-I and I-E synaptic strengths, respectively. For all synapses we used the same decay time constant *τ* = 3.0 *ms*. The reversal potential of the synaptic current ($E_{syn}^{j}$) was set to 0 mV for excitatory synapses and -75mV for inhibitory synapses.

### Generation of heterogenous ACh spatial maps

To simulate spatially heterogeneous distribution of ACh levels, we constructed a mapping of maximal conductance values *g*_*Ks*_ across the E cell and I cell lattices (Fig 9, bottom layer). The $g_{K_{s}}^{i,j}$ values for E cells, based on their *i*, *j* position in the 20×20 lattice, were computed by an iterative process that approximates locally diffusive spread of $g_{K_{s}}$ in response to a point source release with subsequent decay. The iterative process and resulting $g_{K_{s}}$ mapping were computed prior to simulation of neural network activity and remained constant throughout the network simulation. In the iterative process, initially, all $g_{K_{s}}^{i,j}$ values were set to 1.5 mS/cm^2^ and the sites of point source release were chosen.

The value of $g_{K_{s}}^{i,j}$ at iteration step *n*+1 was computed by:

$$g_{K_{s}}^{i,j}(n+1)=D\Delta t\left(\frac{g_{K_{s}}^{i+1,j}(n)-2g_{K_{s}}^{i,j}(n)+g_{K_{s}}^{i-1,j}(n)}{{\left(\Delta x\right)}^{2}}+\frac{g_{K_{s}}^{i,j+1}(n)-2g_{K_{s}}^{i,j}(n)+g_{K_{s}}^{i,j-1}(n)}{{\left(\Delta y\right)}^{2}}\right)-R_{ij}\Delta t+B\Delta t\left(\max g_{K_{s}}-g_{K_{s}}^{ij}(n)\right)+g_{K_{s}}^{ij}(n)$$

where the first term with coefficient *D* as the diffusion constant represented discrete diffusion on the 2D lattice network, *R*_*ij*_ simulated the effect of ACh release (modeled by a decreased $g_{K_{s}}$ level) and *B* was the decay constant to represent the effect of ACh decay. Periodic boundary conditions were imposed for cells at the lattice edges. The iteration time-step was Δ*t*. For simplification we set Δ*t*, Δ*x*, Δ*y* = 1 for unit time step and unit distances on the lattice. The $g_{K_{s}}$ levels for the I cells were assigned as the average value of the 4 nearest E cells in the 2×2 block centered on the I cell. Simulations shown in S1 Fig hold *g*_*Ks*_ fixed at 0 mS/cm^2^ across all I cells, without qualitatively changing results.

In all $g_{K_{s}}$ mappings, we set the lower bound of $g_{K_{s}}$ to be 0.2 mS/cm^2^ and the upper bound to 1.5 mS/cm^2^, if not otherwise specified. The iterative process was frozen at different time steps to yield low $g_{K_{s}}$ (high ACh) regions with different radii. The frozen $g_{K_{s}}$ mappings dictated the $g_{K_{s}}^{i,j}$ values used during the simulations of neural network activity.

### Measurements of dynamics

All results are averages over four simulation replications from random initial conditions, if not specified otherwise. To detect the network-wide oscillatory rhythms, we performed Fourier transforms of the correlogram of the spiking raster plot. We used peak power in the range 2.5−20 Hz to detect theta band power and in the 25−100 Hz range to detect gamma band activity.

We note that gamma oscillations specifically constitute a wide range of frequencies. The specific realization of gamma frequency will depend on specific inhibitory synaptic time-constant used and the drive that the cell receives (internal, coming from the local network, or external from other brain modality).

To detect an individual cell’s rhythmic activity, we performed Fourier transforms of the autocorrelation function of neuronal spiking time-series data. Power in the theta and gamma frequency bands were compared to the average power over all frequencies to identify if a cell exhibited theta or gamma rhythms.

### Local field potentials and coupling strength

To simulate the local field potential (LFP) measurements at different locations in the network, we first convolved the discrete neuronal spiking times with a Gaussian function to generate continuous spike traces for each neuron (V_i_(t) for i^th^ neuron). The Gaussian filter had a σ of 1.5 ms. To compute the LFP at a particular location in the network, spike traces for the E cell at the location and for its 12 nearest neighbor E cells were summed. For the results shown in S13 Fig, we constructed LFP traces by directly summing the voltage traces of these cells.

The LFP trace was filtered at the two frequency ranges determined by the peak frequencies within theta and gamma bands, respectively. We denoted the theta and gamma band filtered LFP signals by x_θ_(t) and x_γ_(t) respectively. The amplitude envelope of x_γ_(t), denoted as A_γ_(t), was extracted using a standard Hilbert transform. To quantify the phase coupling of theta-gamma rhythms, the modulation index (MI) for phase-amplitude coupling between theta and gamma bands was computed as described in [62].

## Supporting information

S1 Fig Network dynamics with no M-current in the I-population. For this simulation, the ***g***_***Ks***_ values of I-population are 0 mS/cm^2^ and E-cells have the same ***g***_***Ks***_ values as in Fig 4H (radius at 6.1 and distance between two spots is 8 units.) The I-I and I-E synaptic strengths were adjusted to 0.06 and 0.035 mS/cm^2^ respectively. **A:** Spike raster plot illustrating E cell (cells 1–400) and I cell (401–500) firing patterns. **B:** Dominant rhythmic activity of individual E-cells (dark blue = none, light blue = theta band, green = gamma band and yellow = mixed, both gamma and theta) plotted at cell position on the E-cell lattice.
(TIF)

S2 Fig Network dynamics with homogeneous *g*_*Ks*_ maps. A: The number of neurons primarily exhibiting gamma (green curve) or theta (blue curve) rhythms as a function of the *g*_*Ks*_ value that is uniform in all cells in the network. B: The rhythm power of network dynamics in theta band (blue curve) and gamma band (green curve) as a function of the network *g*_*Ks*_ value. C, D: Spike raster plots illustrating E cell (cells 1–400) and I cell (401–500) firing patterns with network *g*_*Ks*_ values at 0.2 (C) and 1.2 (D) mS/cm^2^.
(TIF)

S3 Fig Cross-sections of single peak *g*_*Ks*_ distribution. A plot illustrating the cross-sections of *g*_*Ks*_ values with different radii in the simulations of single peak *g*_*Ks*_ distributions; (Fig 3.)
(TIF)

S4 FigGamma and theta band firing varies with g_Ks_ level within a single peak distribution and with neural external drive current.For a single *g*_*Ks*_ hotspot spatial mapping (r = 5.6), the lower bound of *g*_*Ks*_ reached at the center of the hotspot (x-axis, in mS/cm^2^, *g*_*Ks*_ upper bound was set to 1.5 mS/cm^2^) and the external input current to all neurons, $I_{drive}^{i}$ (y-axis) was varied. Panels show measures of network theta power (A), network gamma power (B), ratio of theta to gamma power (C), and numbers of cells primarily firing in the theta frequency band (D), in the gamma frequency band (E) and with power in both bands (mixed, F). Network theta power and the number of cells primarily exhibiting theta rhythmicity were sensitive to level of *g*_*Ks*_ in its spatial distribution. Specifically, for a single *g*_*Ks*_ hotspot with radius r = 5.6 gamma/theta band activity depended on the minimum *g*_*Ks*_ value within the hotspot and the external input current $I_{drive}^{i}$ applied to the neurons. Smaller values for the lower bound of *g*_*Ks*_ increased the difference in neuron modulation within the hotspot compared to outside the hotspot, and larger values of the input current promoted network-wide excitability (i.e. not limited to *g*_*Ks*_ hot spots), leading to global strengthening in theta/gamma power (top/right rows/columns). For dynamics localized to discrete spots of activity (bottom/left-center rows/columns), increased network power in the gamma band, and higher numbers of cells primarily exhibiting gamma activity, occurred for the lower minimum *g*_*Ks*_ values.(TIF)

S5 FigTheta and gamma band frequencies vary with the size of *g*_*Ks*_ hotspot within a single peak distribution.Here the ***g***_***Ks***_ lower bound (i.e. within the hotspot) is ***g***_***Ks***_ = 0.2 mS/cm^2^, while its upper bound (i.e background value) is ***g***_***Ks***_ **= 1.5*mS*/*cm***^**2**^. The external input current to all neurons, $I_{drive}^{i}=3.0\;\mu A/cm^{2}$.(TIF)

S6 Fig Effect of E cell connectivity topology on network dynamics with single peak *g*_*Ks*_ distribution. A, Power of network theta and gamma rhythms as a function of E cell synaptic connection rewiring probability with a single *g*_*Ks*_ hotspot (r = 5.7). It shows the drastic decrease of theta rhythm across the network as network E-cells’ connections became less local. B, The prominent frequency in gamma band as a function of E cell rewiring probability. Inset shows *g*_*Ks*_ spatial mapping on the E cell 2D lattice for the single hotspot (r = 5.7). C and D, Examples of spiking raster plots with E cell rewiring probability at p = 0 and p = 0.875, respectively, demonstrating the shift from theta-gamma coupled activity to a synchronized gamma rhythm. E cells are numbered 1 to 400, and I cells are numbered 401 to 500. Color indicates *g*_*Ks*_ values of cells with the scale in the inset in B. We investigated how network topology affected the observed oscillatory rhythms with a single peak *g*_*Ks*_ spatial distribution. To this effect, we progressively rewired initially local E-E connections to random E cells across the network. The rewiring probability, p, (x-axis on S6A Fig and B) denotes the fraction of E-E connections rewired: when p = 0 the network has the original local excitation/global inhibition connectivity, whereas for p = 1 the network has random excitation spanning the whole network. We observed two major effects as a function of the increased rewiring. First, theta power was significantly diminished for p > 0.1 while gamma power remained relatively high across all rewired connectivities (S6A Fig). This observation underscores the importance of local excitatory connectivity in supporting localized firing within the *g*_*Ks*_ hotspot. Secondly, the frequency of gamma oscillations almost linearly decreased with increasing rewiring (S6B Fig). This is due to the fact, that the random connectivity mediates emergence of zero-phase synchrony between the excitatory neurons. This in turn makes the presynaptic spike arrive on the target excitatory cells at the time when they are (partially) in their refractory times, reducing the impact the EPSP has on these cells. This finally decreases the amount of overall excitation in the network reducing the frequency of gamma. The two rasterplots (S6C and S6D Fig) exemplify these observations.
(TIF)

S7 Fig Changes in network dynamics as a function of M-current time constant, *τ*_*z*_. For the simulations, the double peaked *g*_*Ks*_ spatial mapping is the same as in Fig 4H (the spot radius is r = 6.1 and distance between two spots is d = 8 units.) (A) The frequency in theta band decreased as the M-current time constant, *τ*_*z*_, increased. (B) The frequency in gamma band largely didn’t change as the M-current time constant was increased. (C), (D) Spike raster plots illustrating E cell (cells 1–400) and I cell (401–500) firing patterns when M-current time constant was 25 ms and 125 ms, respectively.
(TIF)

S8 Fig Theta and gamma band frequencies vary with features of a double peaked g_Ks_ spatial distribution. Heatmaps showing the most prominent frequency in the power spectrum of network firing in the theta band (A) and in the gamma band (B) as the radius r of the g_Ks_ hotspots and distance d between hotspot centers is varied. Labels F, G and H correspond to same labels in Fig 4(main). White squares indicate networks without significant power in theta or gamma frequency bands. The frequency of network theta and gamma band activity changed as the radius r and distance d between *g*_*Ks*_ hotspot centers were varied in a double peaked *g*_*Ks*_ spatial distribution (S8 Fig). Theta band activity decreased in frequency as the distance between the hotspot centers increased. This was due to fact that the amount of bleed-over excitation from the active hotspot to the silent hotspot decreased as a function of distance between the hotspots. This bleed-over excitation increased excitatory input to the inactive hotspot, subsequently allowing for faster switching between the hot spots when they were close. Gamma band frequency was approximately inversely correlated to the number of cells in the network predominantly exhibiting gamma firing frequency (compare S8B Fig with Fig 4 in main part). In particular, when many cells fired predominantly at gamma frequency, more E cells recruited their local I cells into the PING inhibitory gating that is signaled globally in the network. This stronger inhibitory gating slowed the release of E cells from inhibition and thus network gamma band activity.
(TIF)

S9 Fig Effect of network topology on theta-gamma coupling in a double peak g_Ks_ distribution. A, Power of network theta band activity decreased with increased probability of rewiring synapses between E cells for a double peak g_Ks_ mapping with hotspot radius r = 5.4 and distance between hotspot centers d = 6. The introduction of E-E synaptic connections between the different hotspots allowed cells to fire at the same time and synchronously. B and C, Spike raster plots for E cell rewiring probability at 0 and 0.875, respectively. E cells are numbered 1 to 400, and I cells are 401 to 500. Color indicates g_Ks_ values of cells with the scale in the inset in A. As in the single peak *g*_*Ks*_ spatial distribution, theta-gamma coupled firing activity was sensitive to changes in the local excitation, global inhibition connectivity structure of the network. In this case, we considered a double peak *g*_*Ks*_ spatial mapping which exhibited strong theta-gamma coupling: (r = 5.4, d = 6) and randomly rewired E-E synapses with varying probability. We observed drastic decreases in network theta power with increased E cell rewiring probability (S9 Fig). As rewiring probability increased, network dynamics changed from theta-modulated gamma band activity occurring in each *g*_*Ks*_ hotspot (rewiring probability at 0; S9B Fig) to synchronized gamma band activity in both *g*_*Ks*_ hotspots (rewiring probablity at 0.875; S9C Fig). This was caused by an increase in E-E connectivity between cells in different hotspots. The resulting additional excitation from the active hotspot enabled cells in the silent hotspot to overcome the global inhibition and fire at the same time as the active hotspot and in synchrony with those cells. Theta-gamma coupled activity was well maintained in the ’small-world’ network regime (when the rewiring probability was small, ~0.2). Generally, localized spots of spiking activity (and thus their switching) was obtained when the spatial extent of excitation was smaller than that of inhibition.
(TIF)

S10 FigNetwork dynamics on homogeneous *g*_*Ks*_ spatial map with two hotspots created by increased constant current drive.A) Illustration of applied current distribution for corresponding neurons on the 20 × 20 E cell lattice. Red color indicates neuros receiving $I_{drive}^{i}=4.5\;\mu A/cm^{2}$ and black color indicates cells receiving $I_{drive}^{i}=3.0\mu A/cm^{2}$. B-I, Spiking raster plots (top panels) and network frequency power spectrums (bottom panels) for homogeneous ***g***_***Ks***_ values (as denoted above the panels) between 0 and 1.4 mS/cm^2^.(TIF)

S11 Fig Variability in network rhythmic activity in randomly generated *g*_*Ks*_ maps. A, B and C: Power of network theta (A) and gamma (B) rhythms and their ratio (C) computed from networks with randomly generated *g*_*Ks*_ spatial mappings when the number of hotspot centers was varied from 1 to 20 (y-axis) and hotspot radius r was varied from 2.8 to 5.4 (x-axis). Positions of hotspot centers were randomly chosen on the excitatory cell lattice. Results were averaged over 4 realizations of the *g*_*Ks*_ mapping with different positions of hotspot centers. The ’star’ marker corresponds to parameters for Fig 5A and 5B in main part and the ’square’ marker corresponds to parameters for Fig 5C and 5D in main part. D, E and F, The relative standard error (RSE) of the power of network theta (D) and gamma (E) rhythms and their ratio (F) computed from the 4 realizations of the randomly generated *g*_*Ks*_ spatial mapping with varying hotspot number (y-axis) and radius (x-axis). To systematically consider spatially random *g*_*Ks*_ distributions, we varied the number of *g*_*Ks*_ hotspots and their radius r, and then generated multiple *g*_*Ks*_ spatial mappings with different locations of hotspot centers. Network power in the theta and gamma bands, as well as theta-gamma power ratio, averaged over simulations from 4 realizations of the *g*_*Ks*_ mapping, varied widely. This was due to high variation in network rhythmic activity generated across the 4 *g*_*Ks*_ mappings realizations. Computation of the relative standard error (RSE) in power of network activity in the theta and gamma frequency bands across the *g*_*Ks*_ mapping realizations showed that gamma band power was generally similar, but theta band power and, thus, theta-gamma power ratio, showed higher variability across mapping realizations.
(TIF)

S12 Fig Theta and gamma band frequencies vary with features of randomly generated *g*_*Ks*_ maps. Heatmaps show the most prominent frequency in the power spectrum of network firing in the theta band (A) and in the gamma band (B) as the number of hotspot centers was varied from 1 to 20 (y-axis) and hotspot radius r was varied from 2.8 to 5.4 (x-axis).
(TIF)

S13 Fig Strength of theta-gamma coupling as a function of the distance from the low *g*_*Ks*_ region. Modulation Index (MI) between gamma and theta filtered LFP traces as a function of the distance from the center of the *g*_*Ks*_ hotspot as in Fig 6. The only difference is here we use the sum of cell voltage traces for the LFP calculation.
(TIF)

S14 FigChanges in theta and gamma frequencies as a function of cellular signaling properties.For these simulations, the double peaked ***g***_***Ks***_ spatial mapping is the same as in Fig 4H (radii r = 6.1 and distance between two spots d = 8 units). A) The theta band frequency increased as the E-cell external current $I_{drive}^{i}$ input was increased. B) The gamma band frequency increased as the E-cell external current $I_{drive}^{i}$ input was increased. C) The theta band frequency increased as the decay time constant for inhibitory synapses ***τ***_***I***_ was increased. D) The gamma band frequency decreased as the decay time constant for inhibitory synapses ***τ***_***I***_ was increased. For C and D we kept the product of inhibitory synaptic strength and ***τ***_***I***_ constant in order to achieve similar inhibition level across the set of simulations.(TIF)

S15 Fig Network dynamics with sparse inhibitory connectivity. For the simulations, the double peaked *g*_*Ks*_ spatial mapping is the same as in Fig 4H (radii is 6.1 and distance between two spots is 8 units.) Spike raster plots illustrating E cell (cells 1–400) and I cell (401–500) firing patterns. A, Default inhibitory synaptic connectivity (all-to-all, 0.04 mS/cm^2^ as inhibitory synaptic strength, detailed in Materials and Methods section). B, Random connectivity with 80 percent of default inhibitory synaptic density, inhibitory synaptic strengths are adjusted to 0.045 mS/cm^2^. C, Random connectivity with 60 percent of default inhibitory synaptic density, inhibitory synaptic strengths are adjusted to 0.048 mS/cm^2^. D, Random connectivity with 40 percent of default inhibitory synaptic density, inhibitory synaptic strengths are adjusted to 0.075 mS/cm^2^.
(TIF)
