# Supplementary figures and images for: Theta-gamma coupling emerges from spatially heterogeneous cholinergic neuromodulation
Source: PLoS Comput Biol. 2021 Jul 30;17(7):e1009235. doi: 10.1371/journal.pcbi.1009235 (PMC8357148; doi:10.1371/journal.pcbi.1009235)

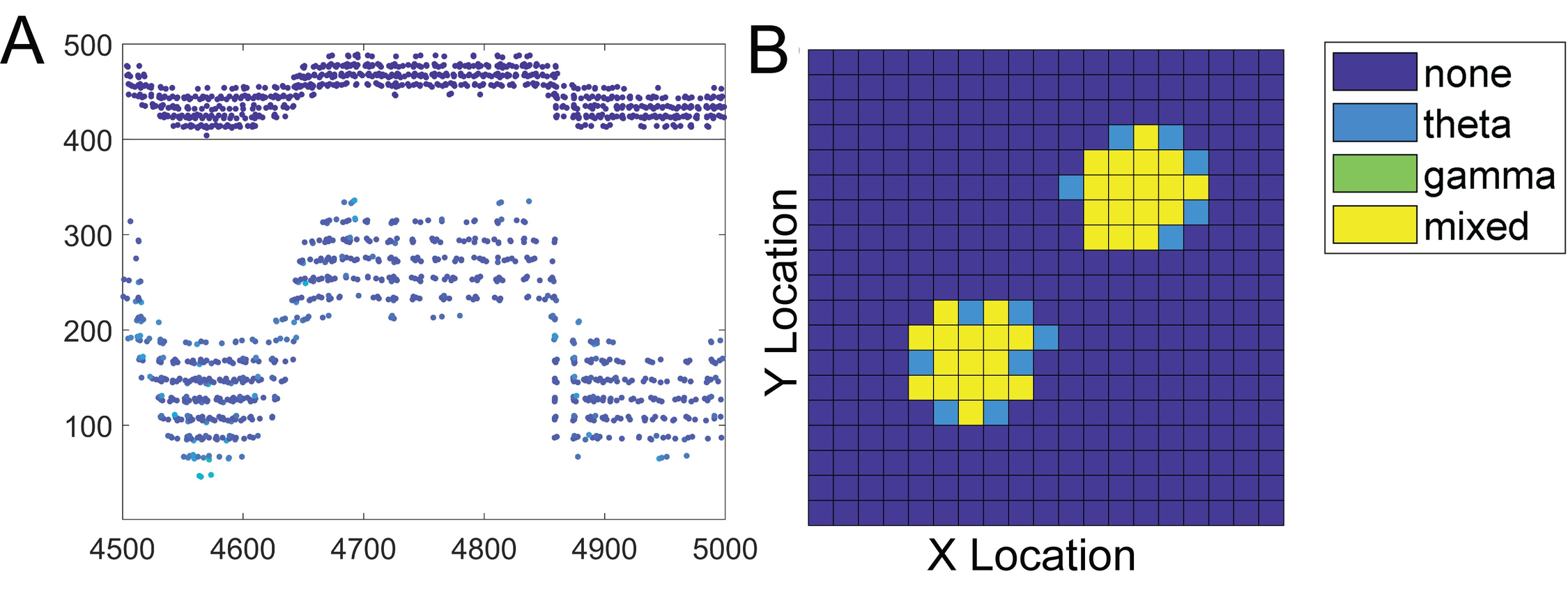

Supplement: S1 Fig — For this simulation, the gKs values of I-population are 0 mS/cm2 and E-cells have the same gKs values as in Fig 4H (radius at 6.1 and distance between two spots is 8 units.) The I-I and I-E synaptic strengths were adjusted to 0.06 and 0.035 mS/cm2 respectively. A: Spike raster plot illustrating E cell (cells 1–400) and I cell (401–500) firing patterns. B: Dominant rhythmic activity of individual E-cells (dark blue = none, light blue = theta band, green = gamma band and yellow = mixed, both gamma and theta) plotted at cell position on the E-cell lattice. (TIF) [file pcbi.1009235.s001.tif]

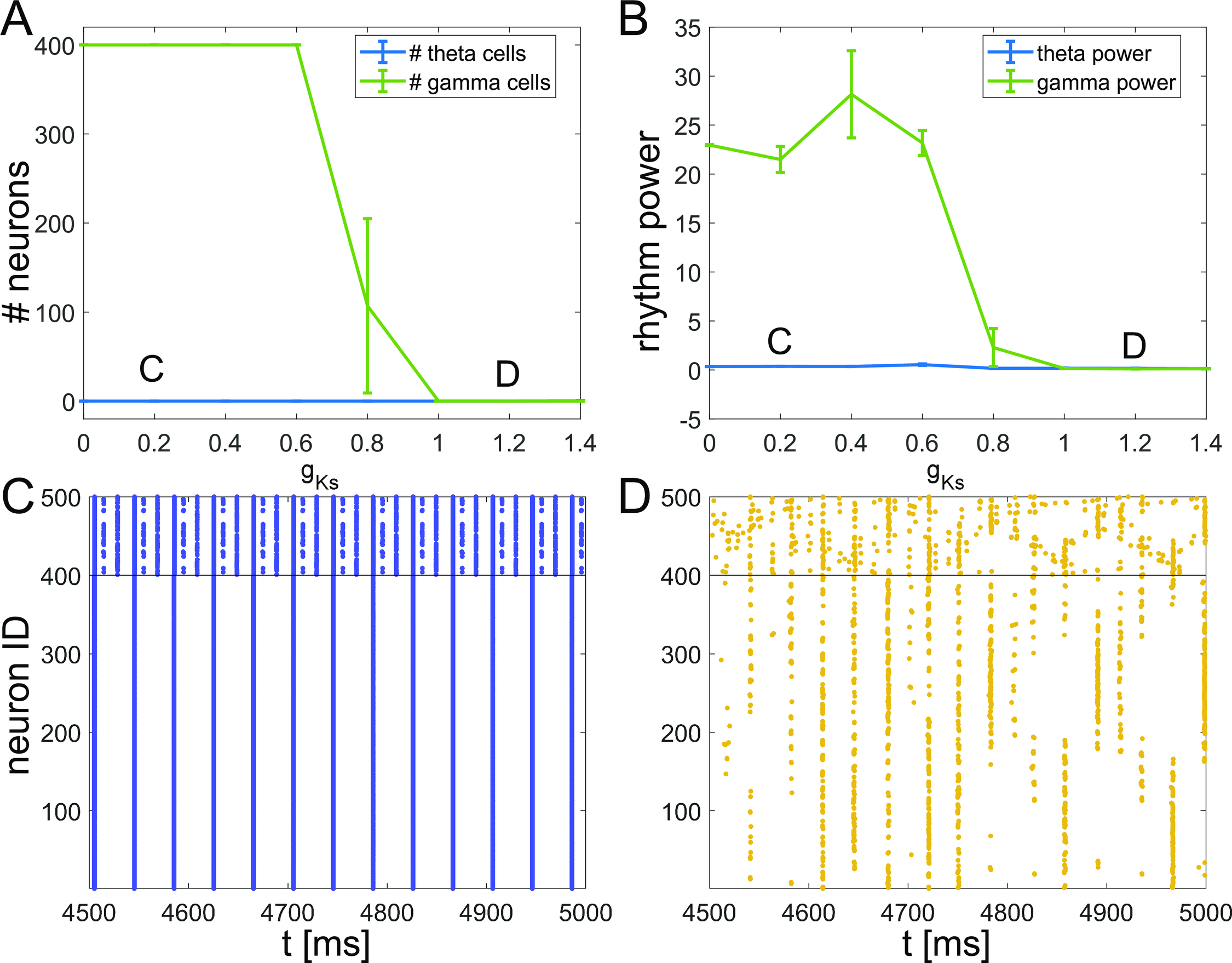

Supplement: S2 Fig — A: The number of neurons primarily exhibiting gamma (green curve) or theta (blue curve) rhythms as a function of the gKs value that is uniform in all cells in the network. B: The rhythm power of network dynamics in theta band (blue curve) and gamma band (green curve) as a function of the network gKs value. C, D: Spike raster plots illustrating E cell (cells 1–400) and I cell (401–500) firing patterns with network gKs values at 0.2 (C) and 1.2 (D) mS/cm2. (TIF) [file pcbi.1009235.s002.tif]

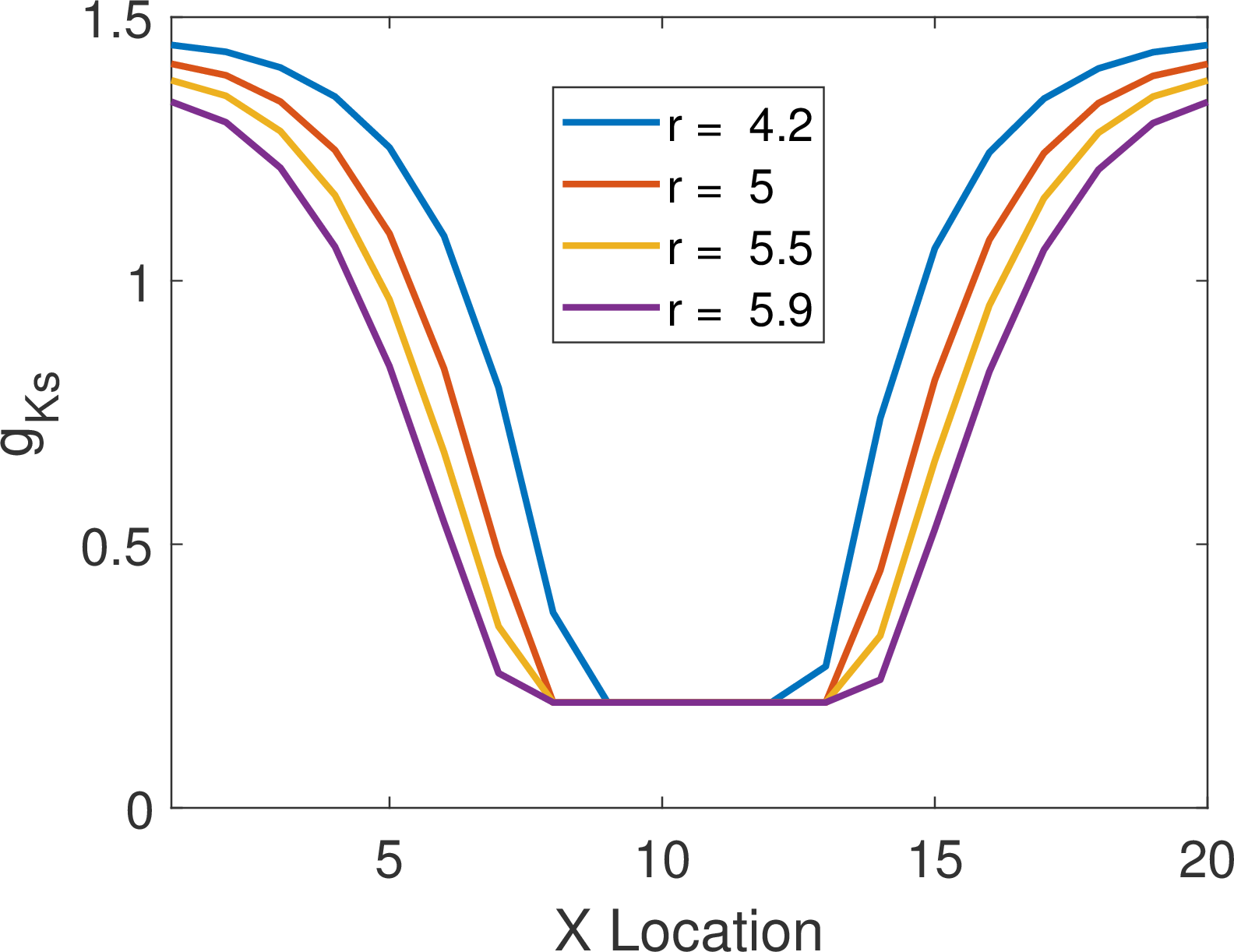

Supplement: S3 Fig — A plot illustrating the cross-sections of gKs values with different radii in the simulations of single peak gKs distributions; (Fig 3.) (TIF) [file pcbi.1009235.s003.tif]

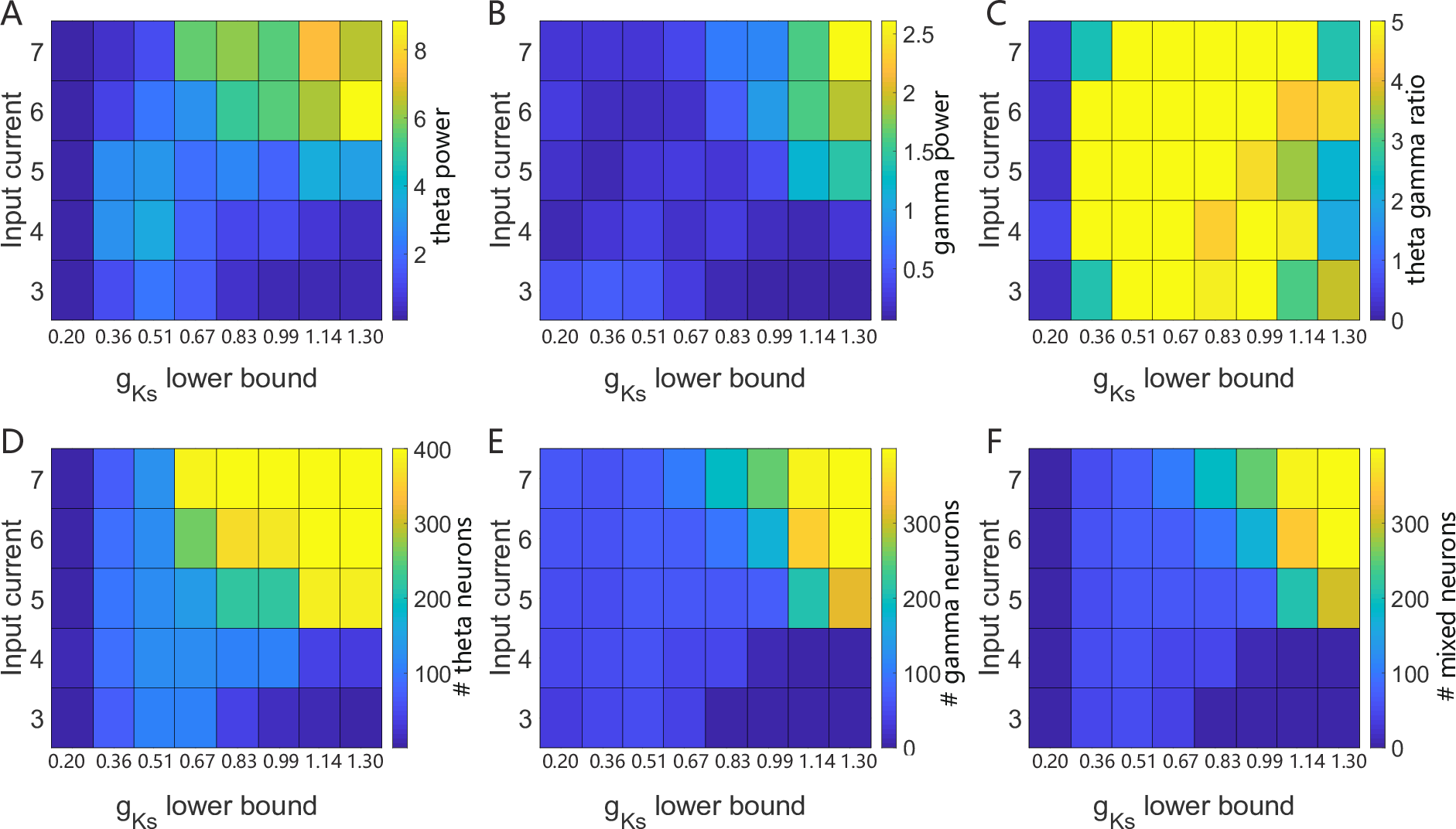

Supplement: S4 Fig — For a single gKs hotspot spatial mapping (r = 5.6), the lower bound of gKs reached at the center of the hotspot (x-axis, in mS/cm2, gKs upper bound was set to 1.5 mS/cm2) and the external input current to all neurons, Idrivei (y-axis) was varied. Panels show measures of network theta power (A), network gamma power (B), ratio of theta to gamma power (C), and numbers of cells primarily firing in the theta frequency band (D), in the gamma frequency band (E) and with power in both bands (mixed, F). Network theta power and the number of cells primarily exhibiting theta rhythmicity were sensitive to level of gKs in its spatial distribution. Specifically, for a single gKs hotspot with radius r = 5.6 gamma/theta band activity depended on the minimum gKs value within the hotspot and the external input current Idrivei applied to the neurons. Smaller values for the lower bound of gKs increased the difference in neuron modulation within the hotspot compared to outside the hotspot, and larger values of the input current promoted network-wide excitability (i.e. not limited to gKs hot spots), leading to global strengthening in theta/gamma power (top/right rows/columns). For dynamics localized to discrete spots of activity (bottom/left-center rows/columns), increased network power in the gamma band, and higher numbers of cells primarily exhibiting gamma activity, occurred for the lower minimum gKs values. (TIF) [file pcbi.1009235.s004.tif]

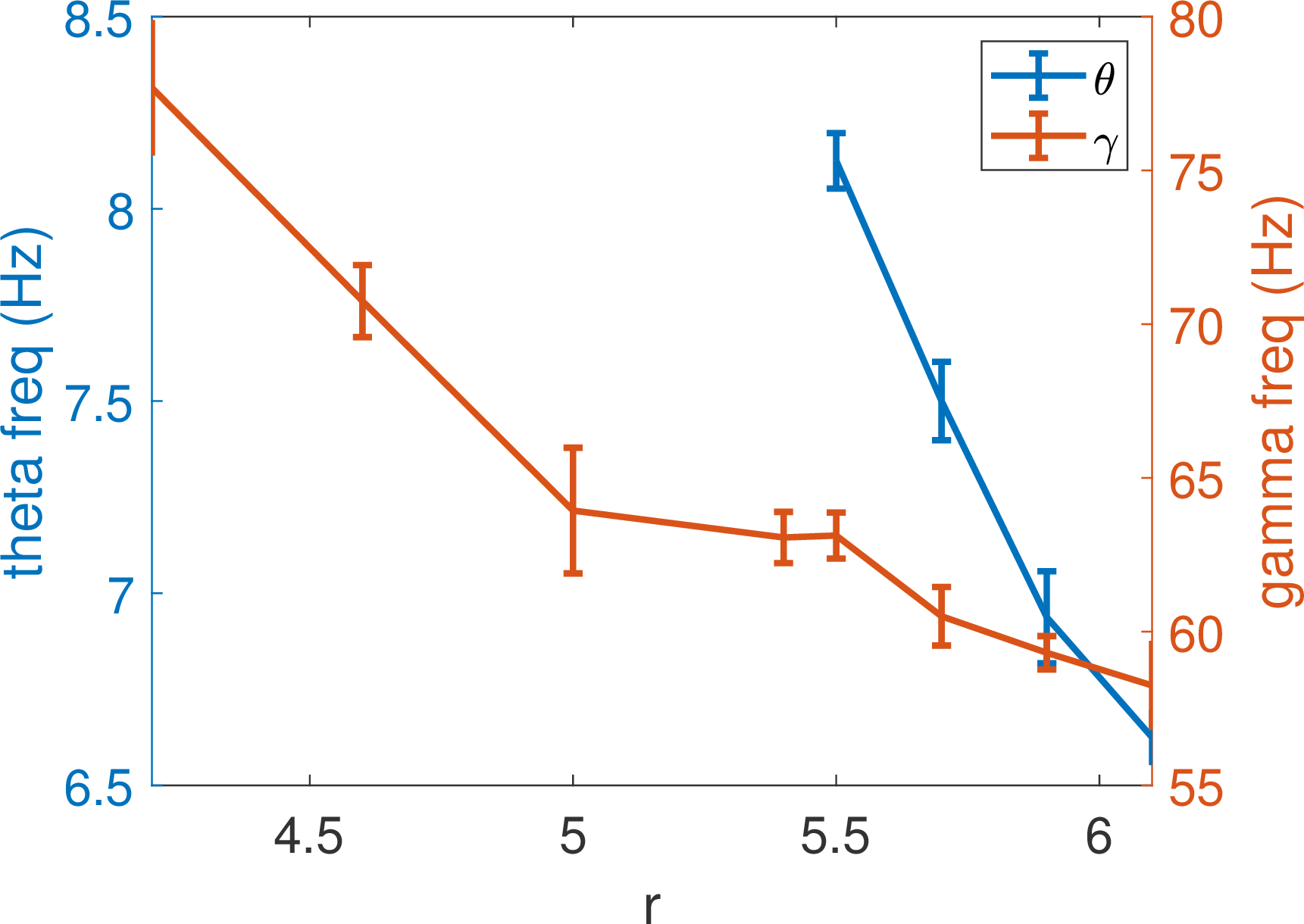

Supplement: S5 Fig — Here the gKs lower bound (i.e. within the hotspot) is gKs = 0.2 mS/cm2, while its upper bound (i.e background value) is gKs = 1.5mS/cm2. The external input current to all neurons, Idrivei=3.0μA/cm2. (TIF) [file pcbi.1009235.s005.tif]

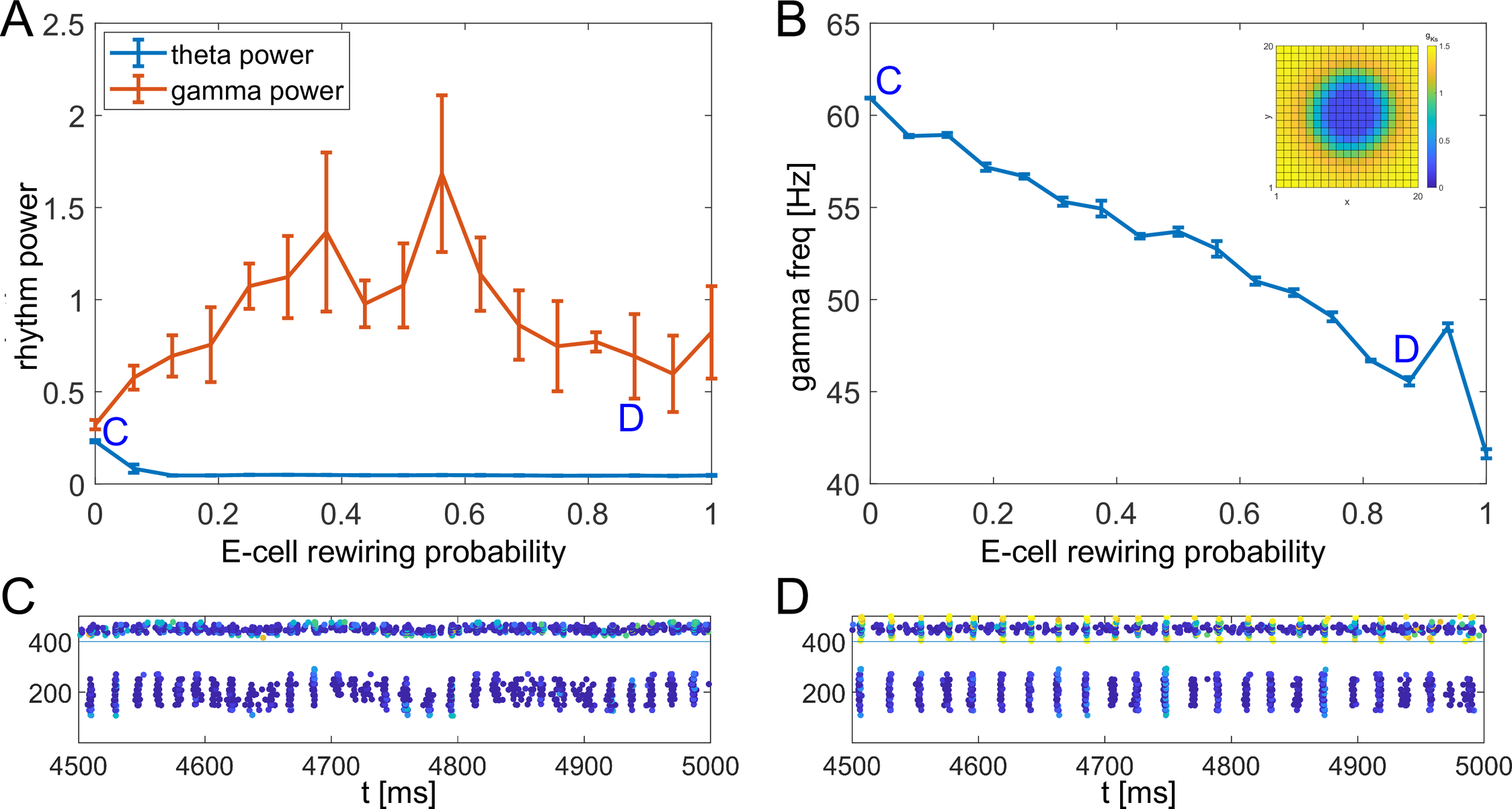

Supplement: S6 Fig — A, Power of network theta and gamma rhythms as a function of E cell synaptic connection rewiring probability with a single gKs hotspot (r = 5.7). It shows the drastic decrease of theta rhythm across the network as network E-cells’ connections became less local. B, The prominent frequency in gamma band as a function of E cell rewiring probability. Inset shows gKs spatial mapping on the E cell 2D lattice for the single hotspot (r = 5.7). C and D, Examples of spiking raster plots with E cell rewiring probability at p = 0 and p = 0.875, respectively, demonstrating the shift from theta-gamma coupled activity to a synchronized gamma rhythm. E cells are numbered 1 to 400, and I cells are numbered 401 to 500. Color indicates gKs values of cells with the scale in the inset in B. We investigated how network topology affected the observed oscillatory rhythms with a single peak gKs spatial distribution. To this effect, we progressively rewired initially local E-E connections to random E cells across the network. The rewiring probability, p, (x-axis on S6A Fig and B) denotes the fraction of E-E connections rewired: when p = 0 the network has the original local excitation/global inhibition connectivity, whereas for p = 1 the network has random excitation spanning the whole network. We observed two major effects as a function of the increased rewiring. First, theta power was significantly diminished for p > 0.1 while gamma power remained relatively high across all rewired connectivities (S6A Fig). This observation underscores the importance of local excitatory connectivity in supporting localized firing within the gKs hotspot. Secondly, the frequency of gamma oscillations almost linearly decreased with increasing rewiring (S6B Fig). This is due to the fact, that the random connectivity mediates emergence of zero-phase synchrony between the excitatory neurons. This in turn makes the presynaptic spike arrive on the target excitatory cells at the time when they are (partially) in the [file pcbi.1009235.s006.tif]

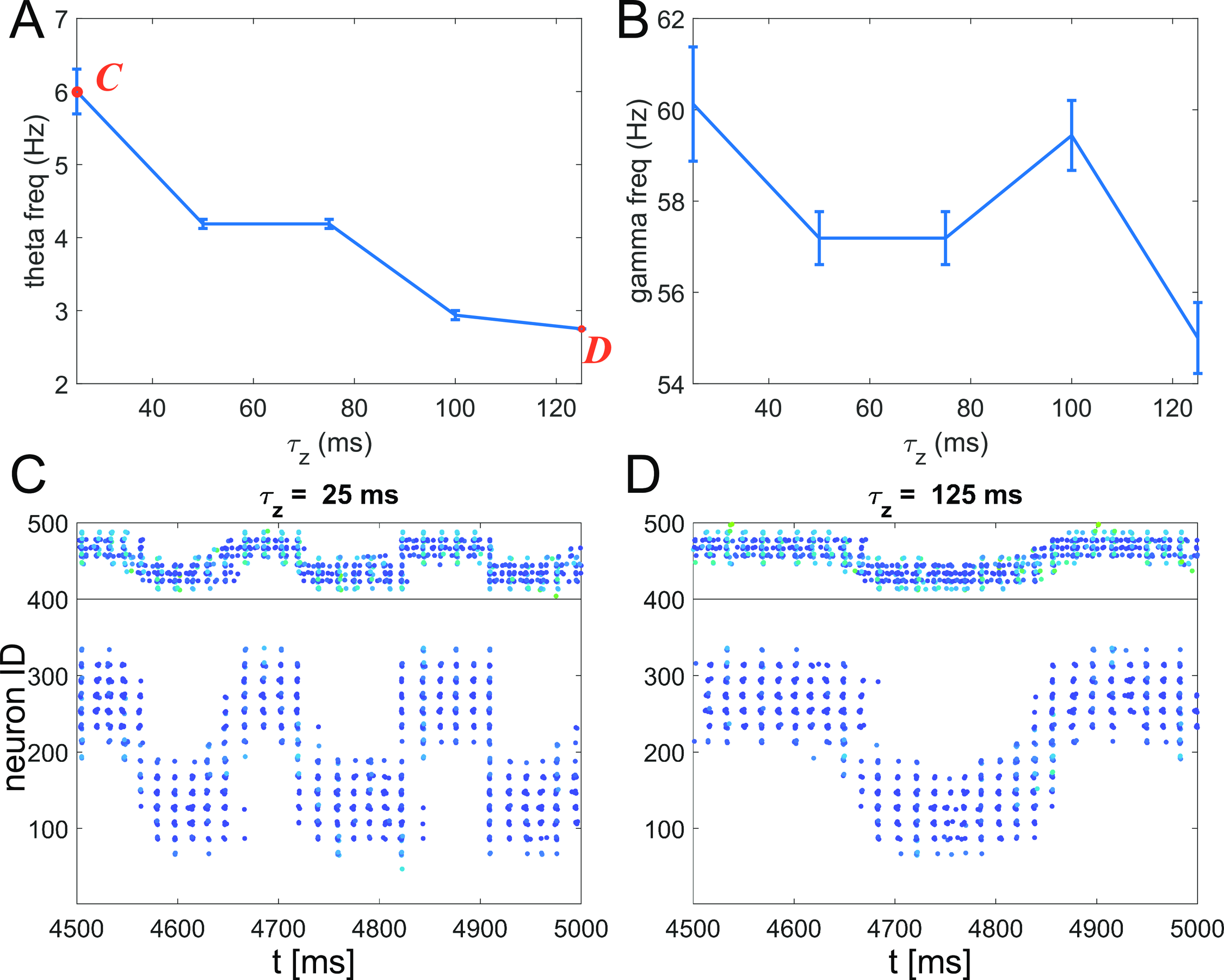

Supplement: S7 Fig — For the simulations, the double peaked gKs spatial mapping is the same as in Fig 4H (the spot radius is r = 6.1 and distance between two spots is d = 8 units.) (A) The frequency in theta band decreased as the M-current time constant, τz, increased. (B) The frequency in gamma band largely didn’t change as the M-current time constant was increased. (C), (D) Spike raster plots illustrating E cell (cells 1–400) and I cell (401–500) firing patterns when M-current time constant was 25 ms and 125 ms, respectively. (TIF) [file pcbi.1009235.s007.tif]

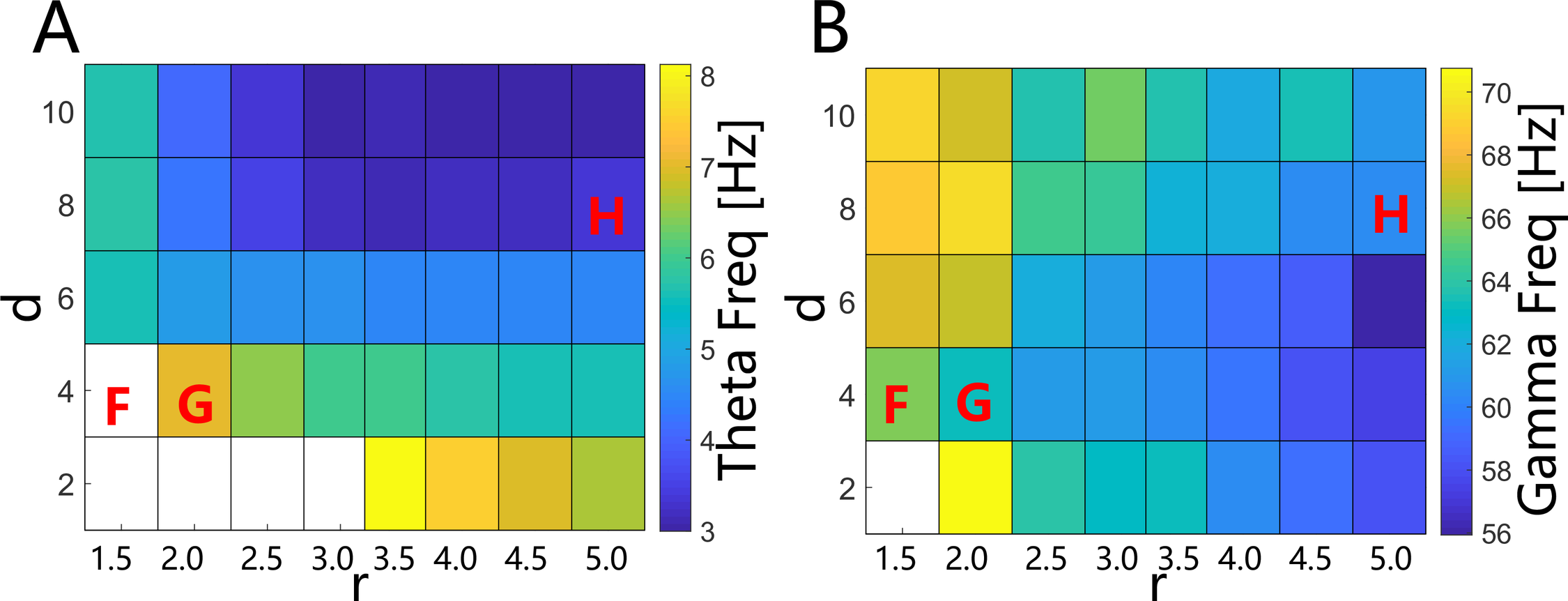

Supplement: S8 Fig — Heatmaps showing the most prominent frequency in the power spectrum of network firing in the theta band (A) and in the gamma band (B) as the radius r of the gKs hotspots and distance d between hotspot centers is varied. Labels F, G and H correspond to same labels in Fig 4(main). White squares indicate networks without significant power in theta or gamma frequency bands. The frequency of network theta and gamma band activity changed as the radius r and distance d between gKs hotspot centers were varied in a double peaked gKs spatial distribution (S8 Fig). Theta band activity decreased in frequency as the distance between the hotspot centers increased. This was due to fact that the amount of bleed-over excitation from the active hotspot to the silent hotspot decreased as a function of distance between the hotspots. This bleed-over excitation increased excitatory input to the inactive hotspot, subsequently allowing for faster switching between the hot spots when they were close. Gamma band frequency was approximately inversely correlated to the number of cells in the network predominantly exhibiting gamma firing frequency (compare S8B Fig with Fig 4 in main part). In particular, when many cells fired predominantly at gamma frequency, more E cells recruited their local I cells into the PING inhibitory gating that is signaled globally in the network. This stronger inhibitory gating slowed the release of E cells from inhibition and thus network gamma band activity. (TIF) [file pcbi.1009235.s008.tif]

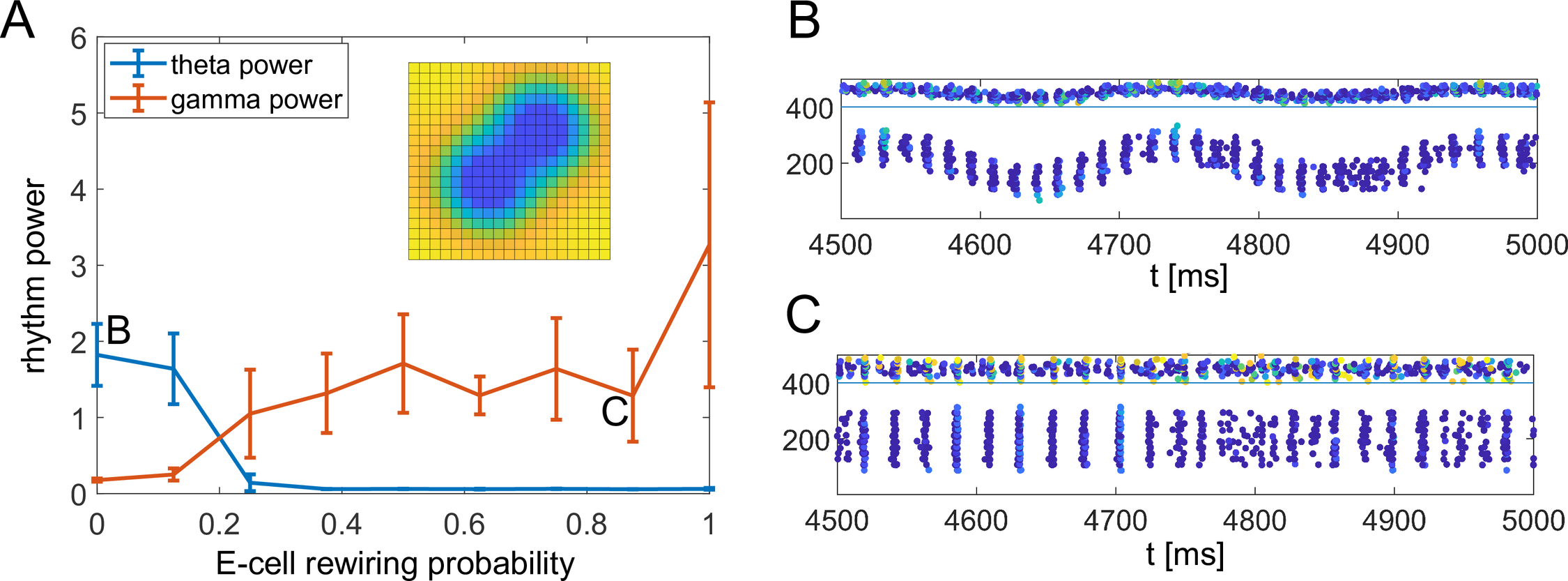

Supplement: S9 Fig — A, Power of network theta band activity decreased with increased probability of rewiring synapses between E cells for a double peak gKs mapping with hotspot radius r = 5.4 and distance between hotspot centers d = 6. The introduction of E-E synaptic connections between the different hotspots allowed cells to fire at the same time and synchronously. B and C, Spike raster plots for E cell rewiring probability at 0 and 0.875, respectively. E cells are numbered 1 to 400, and I cells are 401 to 500. Color indicates gKs values of cells with the scale in the inset in A. As in the single peak gKs spatial distribution, theta-gamma coupled firing activity was sensitive to changes in the local excitation, global inhibition connectivity structure of the network. In this case, we considered a double peak gKs spatial mapping which exhibited strong theta-gamma coupling: (r = 5.4, d = 6) and randomly rewired E-E synapses with varying probability. We observed drastic decreases in network theta power with increased E cell rewiring probability (S9 Fig). As rewiring probability increased, network dynamics changed from theta-modulated gamma band activity occurring in each gKs hotspot (rewiring probability at 0; S9B Fig) to synchronized gamma band activity in both gKs hotspots (rewiring probablity at 0.875; S9C Fig). This was caused by an increase in E-E connectivity between cells in different hotspots. The resulting additional excitation from the active hotspot enabled cells in the silent hotspot to overcome the global inhibition and fire at the same time as the active hotspot and in synchrony with those cells. Theta-gamma coupled activity was well maintained in the ’small-world’ network regime (when the rewiring probability was small, ~0.2). Generally, localized spots of spiking activity (and thus their switching) was obtained when the spatial extent of excitation was smaller than that of inhibition. (TIF) [file pcbi.1009235.s009.tif]

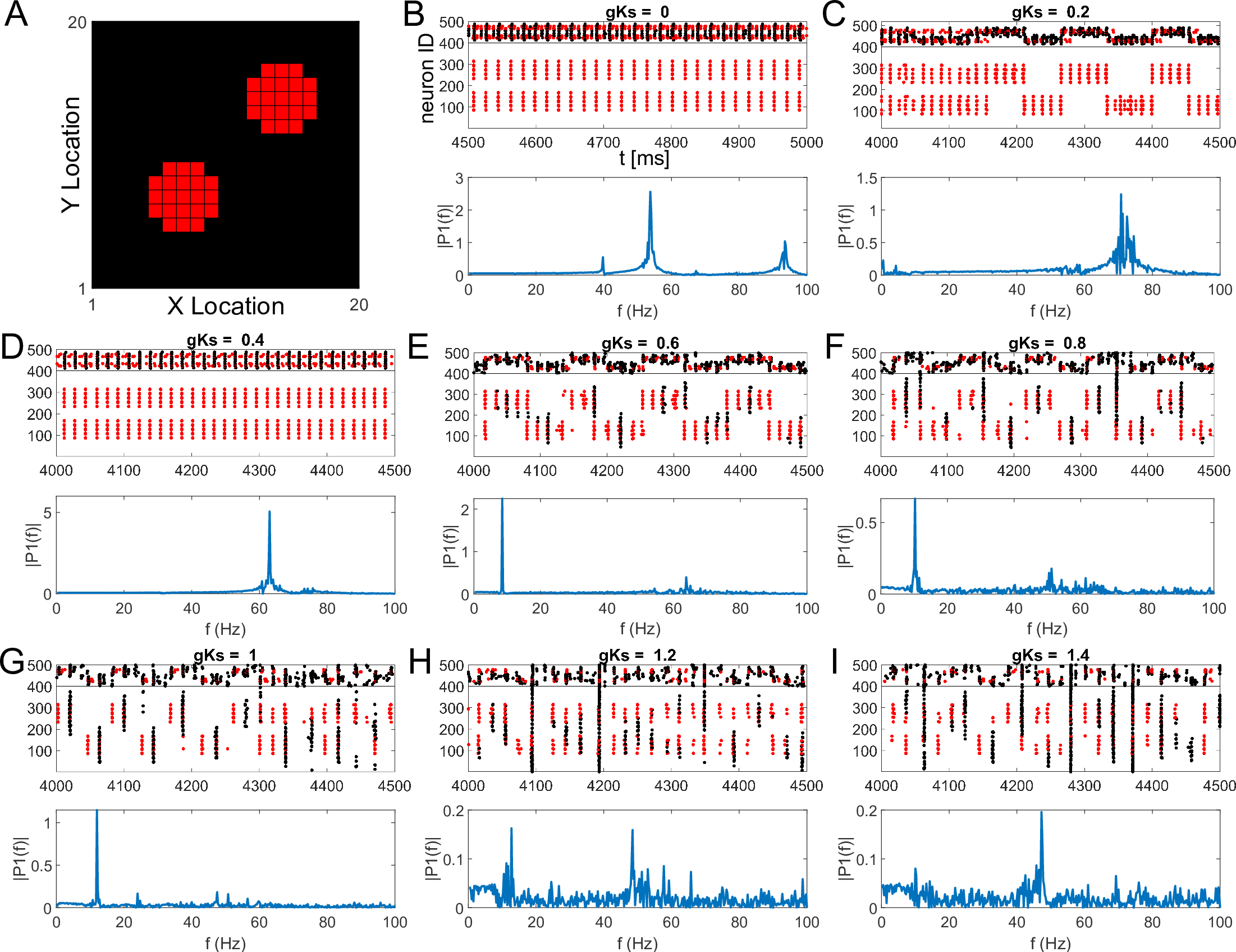

Supplement: S10 Fig — A) Illustration of applied current distribution for corresponding neurons on the 20 × 20 E cell lattice. Red color indicates neuros receiving Idrivei=4.5μA/cm2 and black color indicates cells receiving Idrivei=3.0μA/cm2. B-I, Spiking raster plots (top panels) and network frequency power spectrums (bottom panels) for homogeneous gKs values (as denoted above the panels) between 0 and 1.4 mS/cm2. (TIF) [file pcbi.1009235.s010.tif]

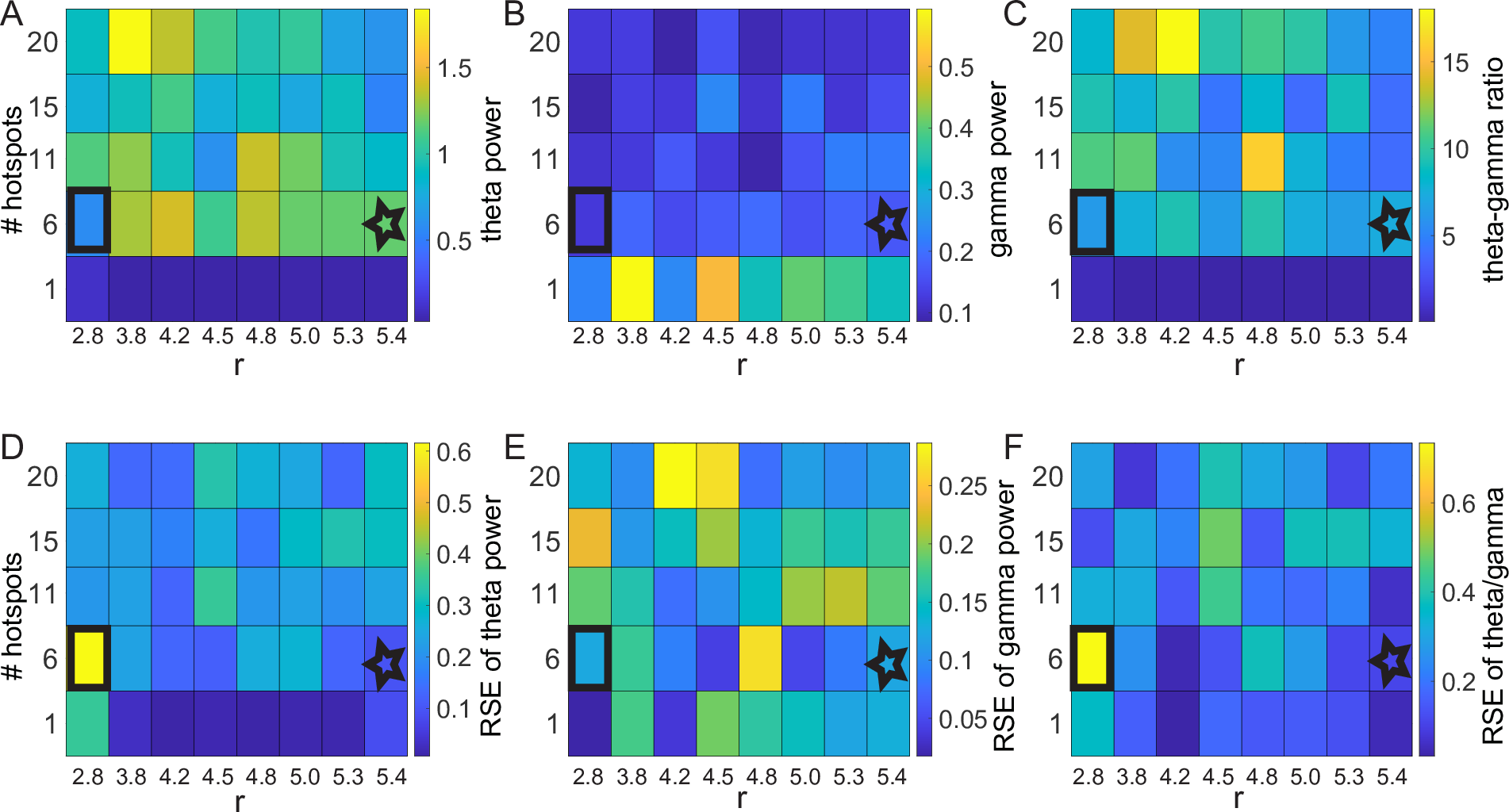

Supplement: S11 Fig — A, B and C: Power of network theta (A) and gamma (B) rhythms and their ratio (C) computed from networks with randomly generated gKs spatial mappings when the number of hotspot centers was varied from 1 to 20 (y-axis) and hotspot radius r was varied from 2.8 to 5.4 (x-axis). Positions of hotspot centers were randomly chosen on the excitatory cell lattice. Results were averaged over 4 realizations of the gKs mapping with different positions of hotspot centers. The ’star’ marker corresponds to parameters for Fig 5A and 5B in main part and the ’square’ marker corresponds to parameters for Fig 5C and 5D in main part. D, E and F, The relative standard error (RSE) of the power of network theta (D) and gamma (E) rhythms and their ratio (F) computed from the 4 realizations of the randomly generated gKs spatial mapping with varying hotspot number (y-axis) and radius (x-axis). To systematically consider spatially random gKs distributions, we varied the number of gKs hotspots and their radius r, and then generated multiple gKs spatial mappings with different locations of hotspot centers. Network power in the theta and gamma bands, as well as theta-gamma power ratio, averaged over simulations from 4 realizations of the gKs mapping, varied widely. This was due to high variation in network rhythmic activity generated across the 4 gKs mappings realizations. Computation of the relative standard error (RSE) in power of network activity in the theta and gamma frequency bands across the gKs mapping realizations showed that gamma band power was generally similar, but theta band power and, thus, theta-gamma power ratio, showed higher variability across mapping realizations. (TIF) [file pcbi.1009235.s011.tif]

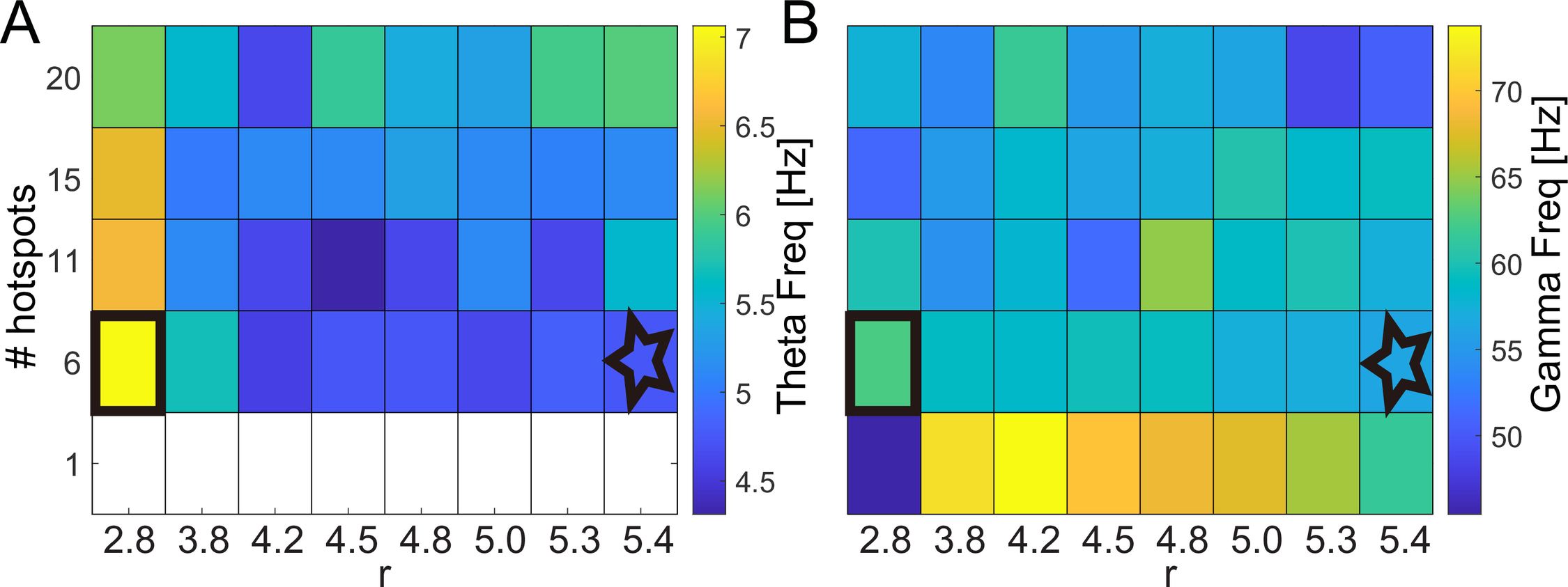

Supplement: S12 Fig — Heatmaps show the most prominent frequency in the power spectrum of network firing in the theta band (A) and in the gamma band (B) as the number of hotspot centers was varied from 1 to 20 (y-axis) and hotspot radius r was varied from 2.8 to 5.4 (x-axis). (TIF) [file pcbi.1009235.s012.tif]

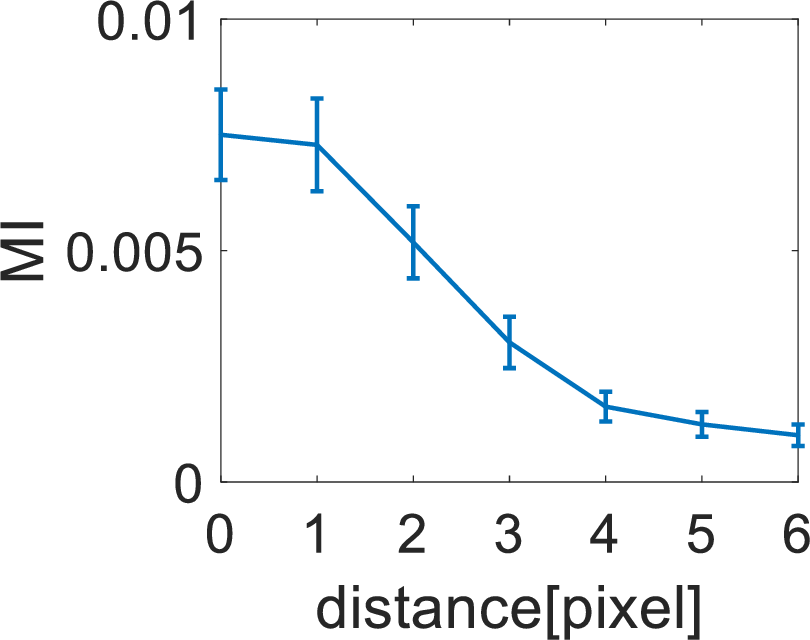

Supplement: S13 Fig — Modulation Index (MI) between gamma and theta filtered LFP traces as a function of the distance from the center of the gKs hotspot as in Fig 6. The only difference is here we use the sum of cell voltage traces for the LFP calculation. (TIF) [file pcbi.1009235.s013.tif]

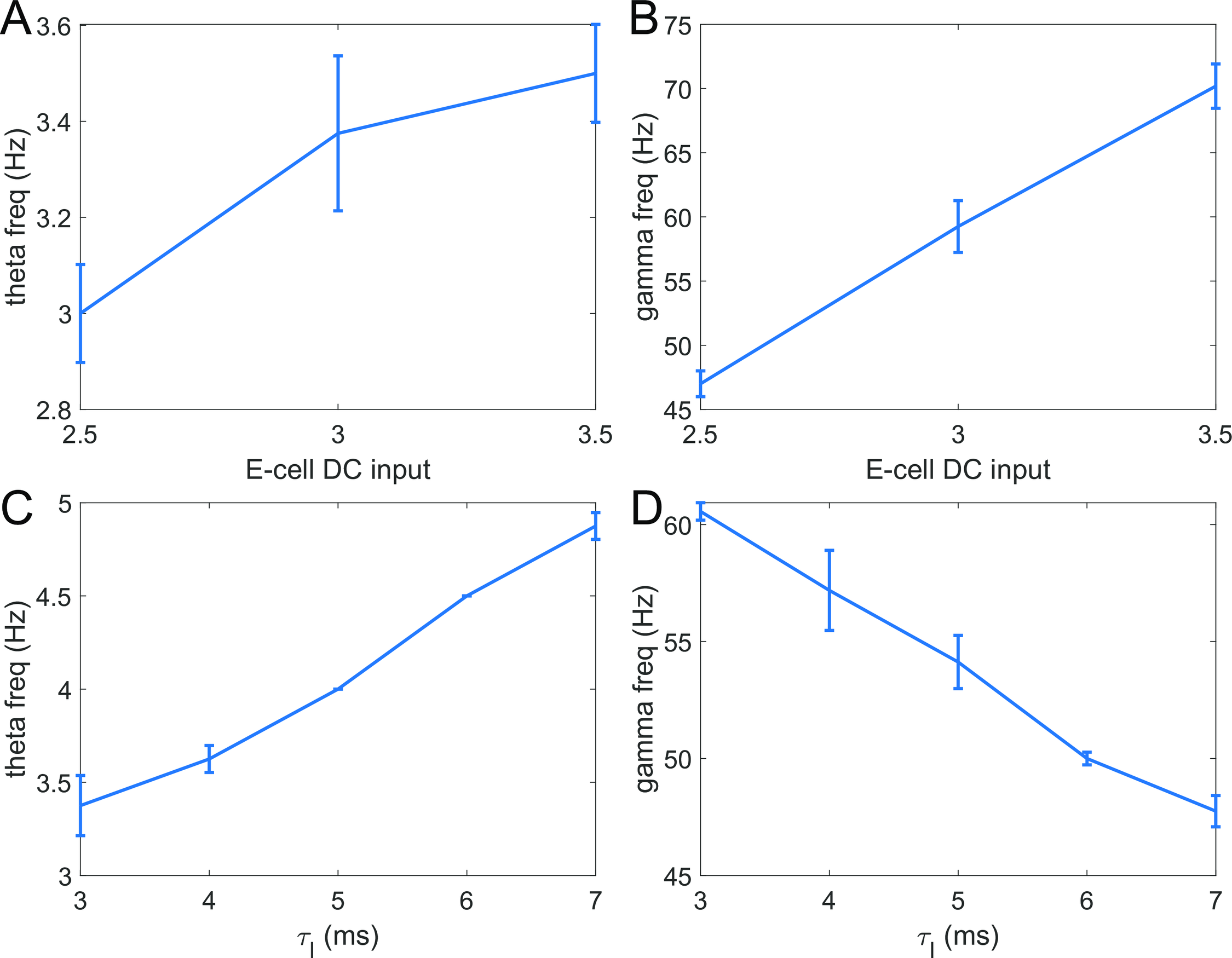

Supplement: S14 Fig — For these simulations, the double peaked gKs spatial mapping is the same as in Fig 4H (radii r = 6.1 and distance between two spots d = 8 units). A) The theta band frequency increased as the E-cell external current Idrivei input was increased. B) The gamma band frequency increased as the E-cell external current Idrivei input was increased. C) The theta band frequency increased as the decay time constant for inhibitory synapses τI was increased. D) The gamma band frequency decreased as the decay time constant for inhibitory synapses τI was increased. For C and D we kept the product of inhibitory synaptic strength and τI constant in order to achieve similar inhibition level across the set of simulations. (TIF) [file pcbi.1009235.s014.tif]

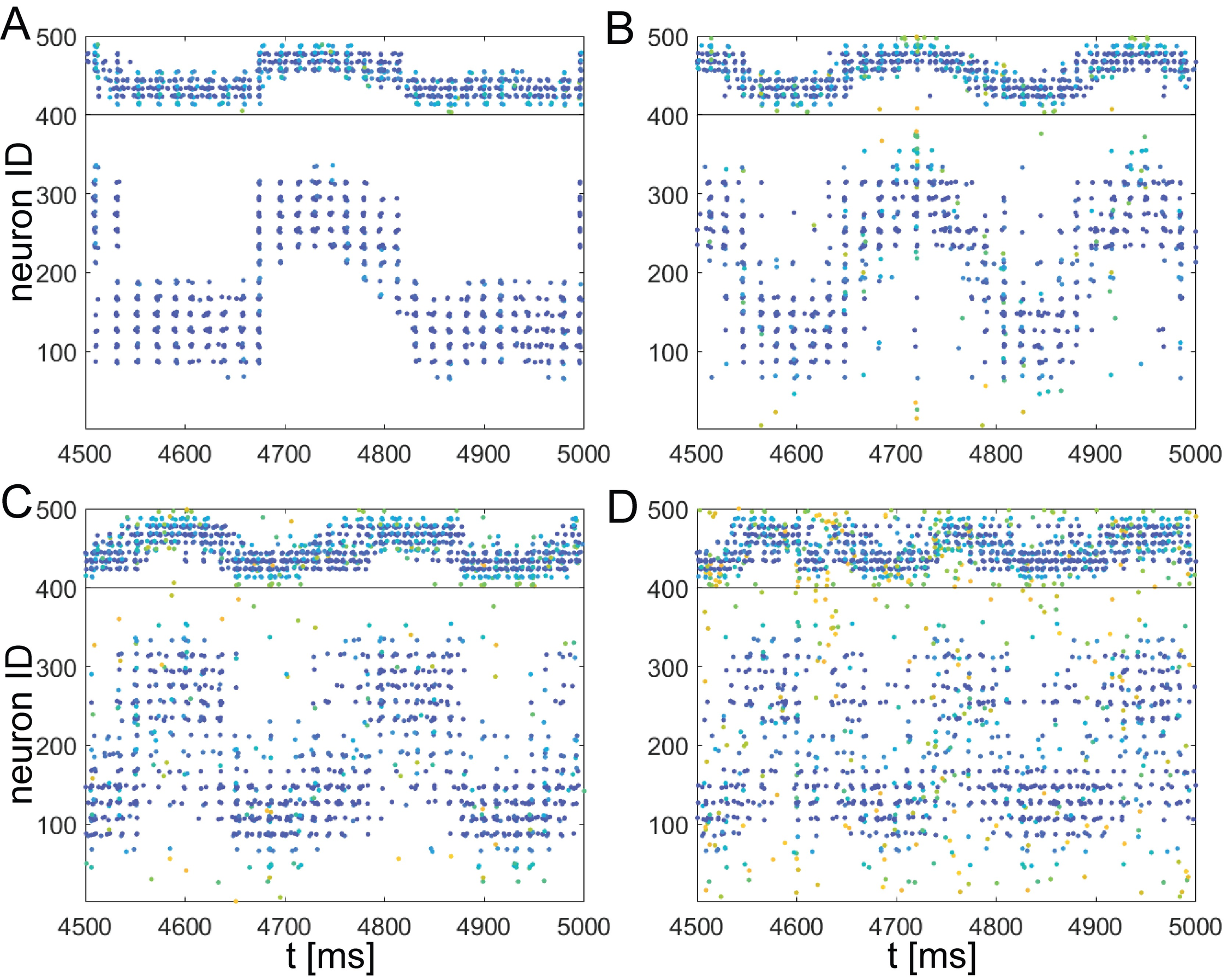

Supplement: S15 Fig — For the simulations, the double peaked gKs spatial mapping is the same as in Fig 4H (radii is 6.1 and distance between two spots is 8 units.) Spike raster plots illustrating E cell (cells 1–400) and I cell (401–500) firing patterns. A, Default inhibitory synaptic connectivity (all-to-all, 0.04 mS/cm2 as inhibitory synaptic strength, detailed in Materials and Methods section). B, Random connectivity with 80 percent of default inhibitory synaptic density, inhibitory synaptic strengths are adjusted to 0.045 mS/cm2. C, Random connectivity with 60 percent of default inhibitory synaptic density, inhibitory synaptic strengths are adjusted to 0.048 mS/cm2. D, Random connectivity with 40 percent of default inhibitory synaptic density, inhibitory synaptic strengths are adjusted to 0.075 mS/cm2. (TIF) [file pcbi.1009235.s015.tif]
